# iPSC-derived CAR-NK extracellular vesicles for non-small cell lung cancer: evidence, engineering, and translational barriers

**DOI:** 10.3389/fimmu.2026.1934758

**Published:** 2026-08-25

**Authors:** Zhanhao Liang, Xutong Zhao, Mingru Jiang, Meizhu Zhou, Shibiao Liu, Yi Zhao

**Affiliations:** 1 Department of Oncology, The First Affiliated Hospital of Dalian Medical University, Dalian, Liaoning, China; 2 Department of Vascular Surgery, Beijing Tsinghua Changgung Hospital, School of Clinical Medicine, Tsinghua Medicine, Tsinghua University, Beijing, China; 3 The First Affiliated Hospital of Dalian Medical University, Dalian, China

**Keywords:** extracellular vesicles, small extracellular vesicles, iPSC-derived NK cells, CAR-NK, NSCLC, inhalation delivery, tumor microenvironment, potency assay

## Abstract

Non-small cell lung cancer (NSCLC) remains difficult to treat with adoptive cell therapies because of antigen heterogeneity, immunosuppressive tumor microenvironments, stromal barriers, and manufacturing variability. Direct evidence for iPSC-derived CAR-NK EV/sEV therapy in NSCLC is currently unavailable. Accordingly, this narrative review evaluates the platform as a translational hypothesis by integrating mechanistic evidence from CAR-engineered EV studies, biological-analog evidence from NK-cell EVs and other engineered EV systems, and clinical-analog evidence from living iPSC-NK or CAR-NK products. The proposed platform could combine a renewable producer-cell source, antigen-directed vesicle binding, and cytotoxic cargo delivery; however, each component, and particularly their integration into a single reproducible product, requires direct experimental validation. Compared with living cell products, CAR-NK sEVs may reduce selected risks related to cellular expansion, persistence, graft-versus-host disease, cytokine release syndrome, and neurotoxicity; however, they do not inherently eliminate antigen-dependent on-target/off-tumor toxicity. Their nanoscale size is hypothesized to improve access to selected stromal barriers in preclinical models, but human solid-tumor penetration remains unproven. Major unresolved issues include rapid systemic clearance and sequestration by hepatic and splenic components of the mononuclear phagocyte system, route-dependent pulmonary deposition, mucus and mucociliary clearance, pulmonary macrophage uptake, subtype- and lesion-specific antigen heterogeneity, EV identity and purity, CAR-positive vesicle quantification, potency-adjusted manufacturing yield, lot comparability, and the absence of potency assays validated across laboratories or linked to clinical outcomes. Thus, iPSC-CAR-NK sEVs should currently be viewed as an investigational translational concept rather than a clinically ready NSCLC therapy.

## 1 Introduction

Lung cancer remains a leading cause of cancer-related mortality, and non-small cell lung cancer (NSCLC) continues to show primary and acquired resistance to immune checkpoint blockade because of antigen heterogeneity, immunosuppressive signaling, stromal barriers, and impaired effector-cell trafficking within the tumor microenvironment (TME) (1–3). Although chimeric antigen receptor T-cell (CAR-T) therapy has established the therapeutic potential of antigen-directed immune-cell engineering, its translation to NSCLC is limited by heterogeneous target expression, restricted tumor infiltration, immunosuppressive tumor microenvironments, manufacturing complexity, and clinically important immune toxicities (4–9). Natural killer (NK) cells provide an alternative effector platform because they can mediate HLA-unrestricted cytotoxicity, support allogeneic manufacturing with a lower intrinsic risk of graft-versus-host disease than allogeneic T-cell products, and retain CAR-independent mechanisms such as antibody-dependent cellular cytotoxicity and stress-ligand recognition (10–13). However, primary NK-cell products remain constrained by donor variability, finite expansion, culture-dependent functional drift, and sensitivity to suppressive cues such as TGF-β, complicating manufacturing standardization and potency control (14–17). Induced pluripotent stem cells (iPSCs) may address selected source-related limitations by providing a renewable clonal platform for standardized CAR-NK producer-cell generation (18–21). Extracellular vesicles (EVs) released by NK cells can contain cytotoxic proteins and immunomodulatory RNAs and are therefore being investigated as acellular effectors or delivery vehicles. In the proposed platform, iPSC-derived CAR-NK cells would serve as standardized producer cells for EV/sEV-enriched preparations that may combine CAR-mediated antigen binding with NK-associated cytotoxic cargo. This cell-free format could reduce selected risks associated with viable-cell expansion and persistence, but it does not inherently eliminate on-target/off-tumor toxicity, rapid systemic clearance, heterogeneous cargo loading, or limited tumor exposure. This narrative review therefore evaluates iPSC-derived CAR-NK EV/sEVs as an investigational translational concept for NSCLC rather than as a clinically established therapeutic platform. Because no product-specific study has directly tested this platform in NSCLC, the review distinguishes mechanistic EV evidence, biological-analog evidence from NK-cell EVs and related engineered EVs, and clinical-analog evidence from living iPSC-NK or CAR-NK products. Claims regarding efficacy, biodistribution, pulmonary delivery, dosing, and safety are consequently treated as hypotheses requiring direct validation.

## 2 Methods

### 2.1 Review design

This article is a narrative review and translational roadmap, not a systematic review, scoping review, or meta-analysis. Because the topic spans several rapidly evolving and heterogeneous fields—including iPSC-derived NK-cell manufacturing, CAR-engineered immune-cell products, extracellular vesicle biology, EV bioprocessing, and early clinical cell-therapy analogs—a formal quantitative synthesis was not appropriate. Accordingly, we did not perform meta-analysis, formal risk-of-bias scoring, or PRISMA-based study flow reporting. Instead, we used a structured narrative approach to identify, classify, and synthesize evidence relevant to the feasibility, limitations, and translational requirements of iPSC-derived CAR-NK EV/sEV products for NSCLC. The structure of this review was informed by principles of transparent reporting for narrative reviews and by EV-specific reporting recommendations, including MISEV2018 and EV-TRACK.

### 2.2 Literature search strategy

A literature search was conducted in PubMed/MEDLINE, Embase, Web of Science Core Collection, and Google Scholar. Additional targeted searches were performed in ClinicalTrials.gov, patent databases, company websites, investor presentations, regulatory or guideline documents, and reference lists of relevant reviews and primary studies. The literature search was updated through July 31, 2026. In addition to the original search terms, targeted searches combined “NSCLC”, “lung adenocarcinoma”, or “lung squamous-cell carcinoma” with “pulmonary immune architecture”, “alveolar macrophage”, “airway mucus”, “mucociliary clearance”, “stromal heterogeneity”, “spatial transcriptomics”, “intratumoral antigen heterogeneity”, “cost of goods”, “techno-economic analysis”, “commercial feasibility”, “regulatory classification”, “potency assay”, “interlaboratory comparison”, “reference standard”, and “relative potency”. To address pharmacokinetic, biodistribution, regulatory, and first-in-human considerations, the updated search additionally combined “extracellular vesicle” or “exosome” with “mononuclear phagocyte system”, “Kupffer cell”, “liver sequestration”, “splenic uptake”, “circulation half-life”, “pharmacokinetics”, “intact-vesicle tracking”, “CD47”, “PEGylation”, “albumin binding”, “macrophage blockade”, “FDA”, “EMA”, “advanced therapy medicinal product”, “biological medicinal product”, “GMP”, “first-in-human”, “MABEL”, “NOAEL”, and “dose escalation”. Regulatory statements were preferentially based on current FDA, EMA, European Commission, ICH, and ISEV documents, whereas non-binding reviews and position papers were used to interpret unresolved EV-specific issues. Studies were prioritized when they provided primary data on lung-compartment-specific biology, regional or subtype-specific target expression, EV manufacturing economics, scale-up comparability, interlaboratory assay performance, or regulatory expectations for potency assurance. For the clinical-development landscape, trial registries, peer-reviewed clinical publications, and sponsor-reported development updates were reviewed through July 31, 2026. Studies were categorized as completed, ongoing, planned development, or no registered interventional study identified. “Ongoing” included studies listed as recruiting or active but not recruiting, whereas “planned development” was reserved for sponsor-announced or regulatory-cleared programs for which participant enrollment had not been confirmed. Observational studies measuring circulating EVs after CAR-T-cell or NK-cell therapy were excluded because they did not administer an EV-based therapeutic product.

### 2.3 Eligibility criteria

We included primary studies, clinical analogs, CMC/manufacturing reports, EV characterization studies, guidelines, and high-relevance reviews. Studies were prioritized if they addressed one or more of the following: CAR-engineered EVs, CAR-NK EVs, NK-cell-derived EVs, iPSC-derived NK cells, NSCLC or solid-tumor delivery models, EV manufacturing and purification, EV identity and purity, potency assays, biodistribution, inhalation delivery, or the safety of CAR- or NK-based products. We excluded studies that were not relevant to EVs, NK cells, CAR engineering, iPSC-derived immune cells, NSCLC, solid-tumor delivery, manufacturing, potency testing, or regulatory translation. We also excluded duplicate reports, articles lacking sufficient methodological detail for interpretation, studies in which the vesicle product was not characterized at least by particle size and EV-associated markers, and studies in which claims could not be traced to a primary source. Non-English articles were considered only when an English abstract or sufficient English-language data were available for accurate interpretation.

### 2.4 Evidence grading and source handling

Product-specific direct evidence was defined as studies testing iPSC-derived CAR-NK EV/sEV products in NSCLC; no such studies were identified. Mechanistic EV evidence included CAR-engineered EV or CAR-NK EV studies demonstrating antigen-dependent binding, uptake, cytotoxicity, or tumor control in other producer-cell or tumor contexts. Biological-analog evidence included NK-cell-derived EVs and other engineered EV systems that informed cargo biology, delivery, biodistribution, or potency. Clinical-analog evidence from living iPSC-NK or CAR-NK therapies was used only to contextualize producer-cell manufacturing, engineering feasibility, dose-scale awareness, repeat-dose logistics, and selected safety endpoints (22, 23). For each evidence domain, we critically assessed the relevance of the experimental model, the adequacy of EV characterization, comparator selection, dose normalization, reproducibility, and whether mechanistic claims were supported by antigen-blockade, loss-of-function, or matched control experiments (24). Apparent discrepancies between studies were interpreted in light of differences in producer-cell source, culture conditions, EV isolation and purification, labeling strategy, administration route, and tumor model rather than being assumed to represent true biological conflict (25). Speculative extrapolations, including assumptions about EV module transfer, stromal access, inhalation stability, dose scaling, and tumor exposure, were identified as such. For quantitative claims, we recorded the product type, model or population, route, dose unit and denominator, source type, and primary citation. Cross-platform doses or efficacy data were not treated as transferable to iPSC-CAR-NK EV/sEV products. Conference abstracts, corporate communications, investor materials, patents, and trial registries were used only when peer-reviewed data were unavailable or when they clarified product design, trial status, manufacturing strategy, dose, or data cutoff dates; they were not treated as direct evidence of efficacy or safety.

### 2.5 Handling of EV terminology and characterization

**Figure 1:**
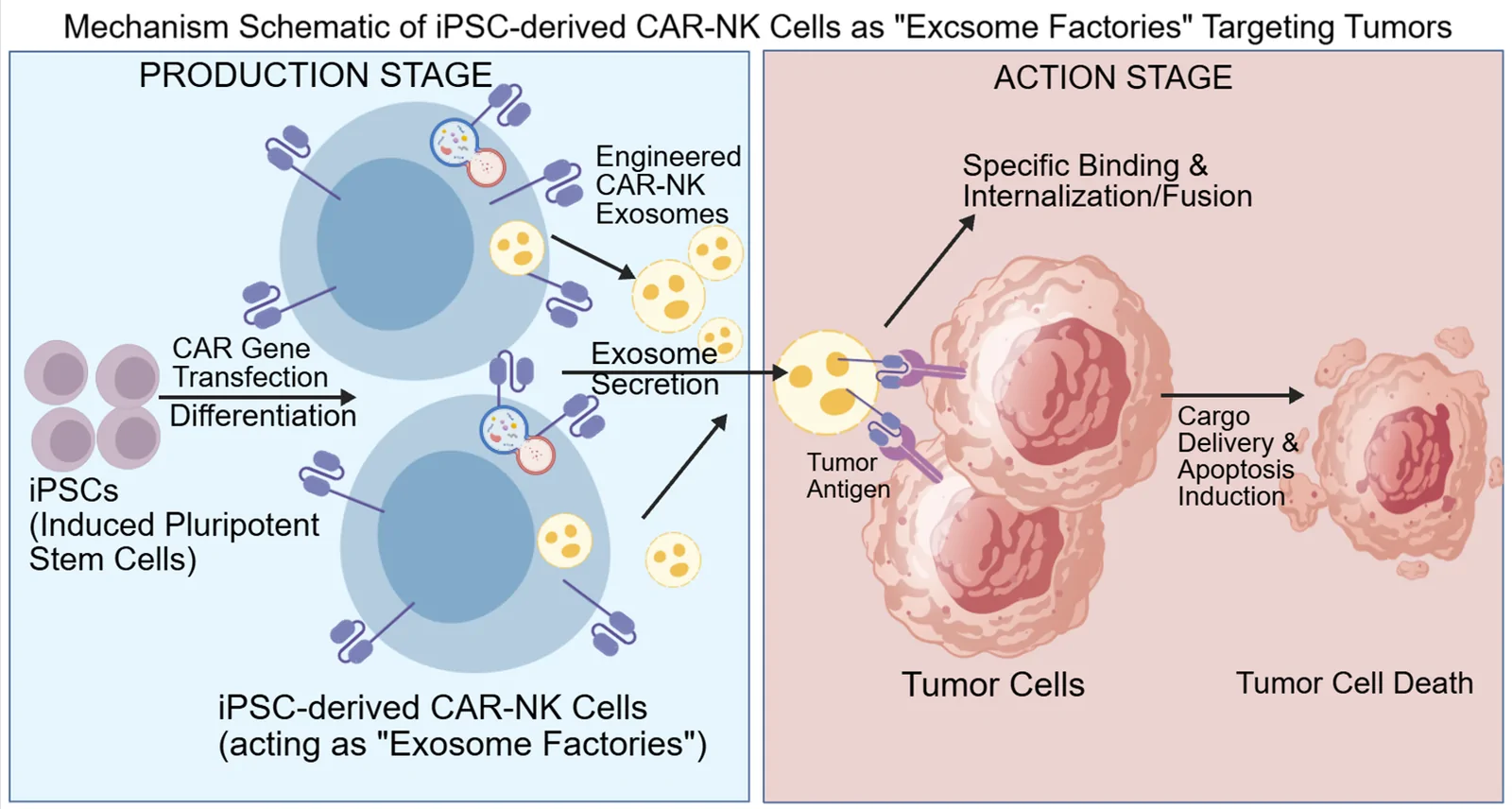
Proposed workflow for generating and evaluating iPSC-derived CAR-NK EV/sEV products. Schematic overview of a two-stage mechanism. Proposed production stage: iPSCs would be engineered with a CAR construct and differentiated into CAR-NK producer cells. Whether the resulting EV/sEV-enriched preparations consistently display CAR and contain functionally relevant cytotoxic cargo remains to be demonstrated directly for iPSC-derived CAR-NK products. Proposed action stage: CAR-positive vesicles may bind antigen-positive tumor cells and deliver vesicular cargo after cellular uptake or membrane interaction; however, the efficiency, mechanism, and *in vivo* relevance of these processes remain unproven in NSCLC. Here, the CAR primarily serves as a targeting ligand to enhance tumor binding/uptake of exosomes; canonical CAR signaling domains are not expected to signal within the acellular vesicle. This concept links an iPSC-based scalable manufacturing platform with nanoscale vesicle-mediated immunotherapy for solid tumors. Vesicle identity should be interpreted operationally as EV/sEV enrichment unless endosomal origin is experimentally demonstrated. Created with BioGDP.com.

EV terminology and reporting were standardized according to MISEV2023, while MISEV2018 was cited only when describing the historical framework used by earlier studies. “Extracellular vesicle” was used as the generic term, and “small EV” or “sEV” was used when vesicle preparations were operationally defined by size distribution, isolation method, density, and marker profile. The term “exosome” was reserved for studies demonstrating endosomal origin. When original articles used “exosome” without direct evidence of endosomal biogenesis, we described the product as EVs or sEVs in this review while noting the terminology used by the source authors when necessary. For EV studies, we extracted available information on source cell, culture conditions, isolation method, particle size, particle concentration, EV-associated markers, negative or depleted markers, cargo characterization, potency assays, and *in vivo* biodistribution or safety testing (26–28) (**Figure 1**).

## 3 Rationale for combining iPSC standardization, CAR targeting, and EV/sEV delivery

Current immunotherapy for non-small cell lung cancer (NSCLC) faces three fundamental barriers: the heterogeneity of cellular products (29), tumor antigen escape (30, 31), and the physical exclusion of immune effectors by the dense tumor stroma (4, 32). Single-modality approaches often fail to address these interconnected challenges simultaneously (33). The proposed platform combines three conceptually distinct elements: iPSC-based producer-cell standardization, CAR-mediated surface targeting, and EV/sEV-based cargo delivery. Evidence supports the feasibility of each element separately, but no study has established that their combination produces a reproducible, tumor-targeted, and therapeutically active iPSC-CAR-NK EV/sEV product in NSCLC (**Table 1**).

**Table 1:** Comparative profile of CAR- and NK-based cellular and extracellular therapeutic platforms.

Platform	Therapeutic format and mechanism	Principal advantages	Key limitations	Evidence maturity
CAR-T cells	Living T cells; CAR ligation activates intracellular signaling, proliferation, cytokine release, and cytotoxicity	Clinically validated CAR targeting; robust expansion and persistence; established regulatory experience	Complex autologous manufacture; CRS, ICANS, and prolonged cytopenias; possible GvHD with allogeneic products; antigen escape, on-target/off-tumor toxicity, and poor solid-tumor infiltration	Approved in hematologic malignancies; investigational in NSCLC
CAR-NK cells	Living NK cells combining CAR-directed recognition with innate cytotoxicity, death-receptor signaling, and potential ADCC	Off-the-shelf potential; CAR-independent tumor recognition; early studies suggest lower CRS, ICANS, and GvHD burden than CAR-T therapy	Limited persistence; source- and process-dependent potency; sensitivity to suppressive TME signals; immune rejection and on-target/off-tumor toxicity remain possible	Early clinical development, mainly in hematologic malignancies
NK-derived EVs	Acellular vesicles carrying variable NK-associated proteins, lipids, and regulatory RNAs	No proliferation or engraftment; potential repeat dosing and cargo loading; retains selected NK cytotoxic and immunomodulatory functions	No engineered antigen specificity; heterogeneous cargo and active-vesicle fraction; rapid hepatic, splenic, and macrophage clearance; uncertain dose and potency standards	Preclinical; no therapeutic clinical study identified
CAR-engineered EVs	EVs displaying CAR-derived binding domains; biological activity depends on antigen engagement and vesicular cargo delivery	Antigen-associated binding without viable-cell expansion; modular surface and cargo engineering	Canonical CAR signaling is absent; variable CAR-positive fraction and CAR density; limited tumor exposure, heterogeneous cargo, and unresolved potency assays; on-target/off-tumor binding remains possible	Preclinical mechanistic evidence; no therapeutic clinical study identified
iPSC-derived CAR-NK EVs	Proposed EV product from clonally engineered iPSC-CAR-NK producer cells, integrating CAR-mediated binding with NK-associated cargo	Renewable producer-cell source; potential reduction in donor variability; multiplex engineering and off-the-shelf manufacture; acellular format	No direct evidence in NSCLC; clonal sourcing does not ensure homogeneous EVs, CAR display, cargo loading, or potency; unresolved clearance, biodistribution, immunogenicity, scalability, cost, and regulatory requirements	Investigational concept supported by indirect EV and living-cell analog evidence

The comparison is qualitative and is not based on matched head-to-head studies. Potential advantages are platform-level hypotheses and should not be interpreted as established superiority. In CAR-engineered EVs, the CAR primarily functions as an antigen-binding module because canonical intracellular CAR signaling is not expected in an acellular vesicle. Nanoscale size does not establish clinically sufficient tumor penetration or accumulation. No direct product-specific evidence is currently available for iPSC-derived CAR-NK EVs in NSCLC. EV terminology follows MISEV2023. ADCC, antibody-dependent cellular cytotoxicity; CAR, chimeric antigen receptor; CRS, cytokine release syndrome; EV, extracellular vesicle; GvHD, graft-versus-host disease; ICANS, immune effector cell-associated neurotoxicity syndrome; iPSC, induced pluripotent stem cell; NK, natural killer cell; NSCLC, non-small cell lung cancer; TME, tumor microenvironment.

### 3.1 Resolving donor heterogeneity via clonal iPSC platforms

Traditional adoptive cell therapies (ACTs) rely heavily on donor-derived sources, such as peripheral blood or cord blood, which inevitably lead to substantial batch-to-batch variability and limit industrial scalability (34). iPSC technology may reduce this bottleneck by providing a renewable clonal source. (1) Clonal consistency and indefinite expansion: Unlike primary cells, iPSCs possess indefinite proliferative capacity (35). A characterized iPSC clone can serve as a master cell bank for large-scale NK-cell differentiation (36). (2) Cellular rejuvenation: The reprogramming process resets telomere length, endowing differentiation-derived NK cells with a “youthful” phenotype that exhibits superior expansion potential and resistance to exhaustion compared to adult-derived counterparts (37). (3) Clinical-analog support for producer-cell manufacturing: Early-phase studies of living iPSC-derived NK or CAR-NK products support the feasibility of clonal, off-the-shelf cell manufacturing. However, these studies do not validate EV yield, vesicle composition, CAR incorporation, potency, pharmacokinetics, or safety and should therefore be interpreted only as manufacturing and clinical-development analogs for an EV product (23).

### 3.2 Precision engineering: iPSCs provide a programmable chassis

While NK cells possess innate anti-tumor activity, relying solely on natural receptors is often insufficient in the complex, immunosuppressive microenvironment of lung cancer. iPSCs serve as a versatile, programmable chassis for precision genetic engineering.(1) Multiplex editing capabilities: Unlike primary NK cells, which are notoriously difficult to transduce efficiently (38), iPSCs can be genetically edited with high precision using CRISPR/Cas9 or other tools. Researchers can introduce multiple functional modules simultaneously, such as tumor-specific chimeric antigen receptors (CARs) targeting antigens such as EGFR or mesothelin, high-affinity non-cleavable CD16 (hnCD16) to enhance antibody-dependent cellular cytotoxicity (ADCC), and autonomous cytokine support, such as IL-15/IL-15Rα (39). (2) Stable inheritance: Stable genetic modification of iPSCs ensures inheritance by the differentiated NK producer cells, but it does not ensure proportional or functional transfer of the engineered module to secreted EVs. Surface display, luminal packaging, abundance, and bioactivity must therefore be quantified directly for each construct and manufacturing condition—while other edits may affect EVs indirectly via altered producer-cell state and vesicle biogenesis. If CAR display is retained on EVs, vesicles may acquire antigen-directed binding capacity, whereas canonical CAR signaling is unlikely to operate in the acellular vesicle context. (3) Resistance to suppression: The iPSC platform allows for the preemptive deletion of inhibitory checkpoints. For example, knocking out the TGF-β receptor (TGFBR2) or the cytokine checkpoint CISH renders the progeny NK cells resistant to the immunosuppressive signaling prevalent in the lung tumor microenvironment, a strategy that is logistically prohibitive with autologous cells (40, 41).

### 3.3 Small EVs may improve access to selected stromal barriers

**Figure 2:**
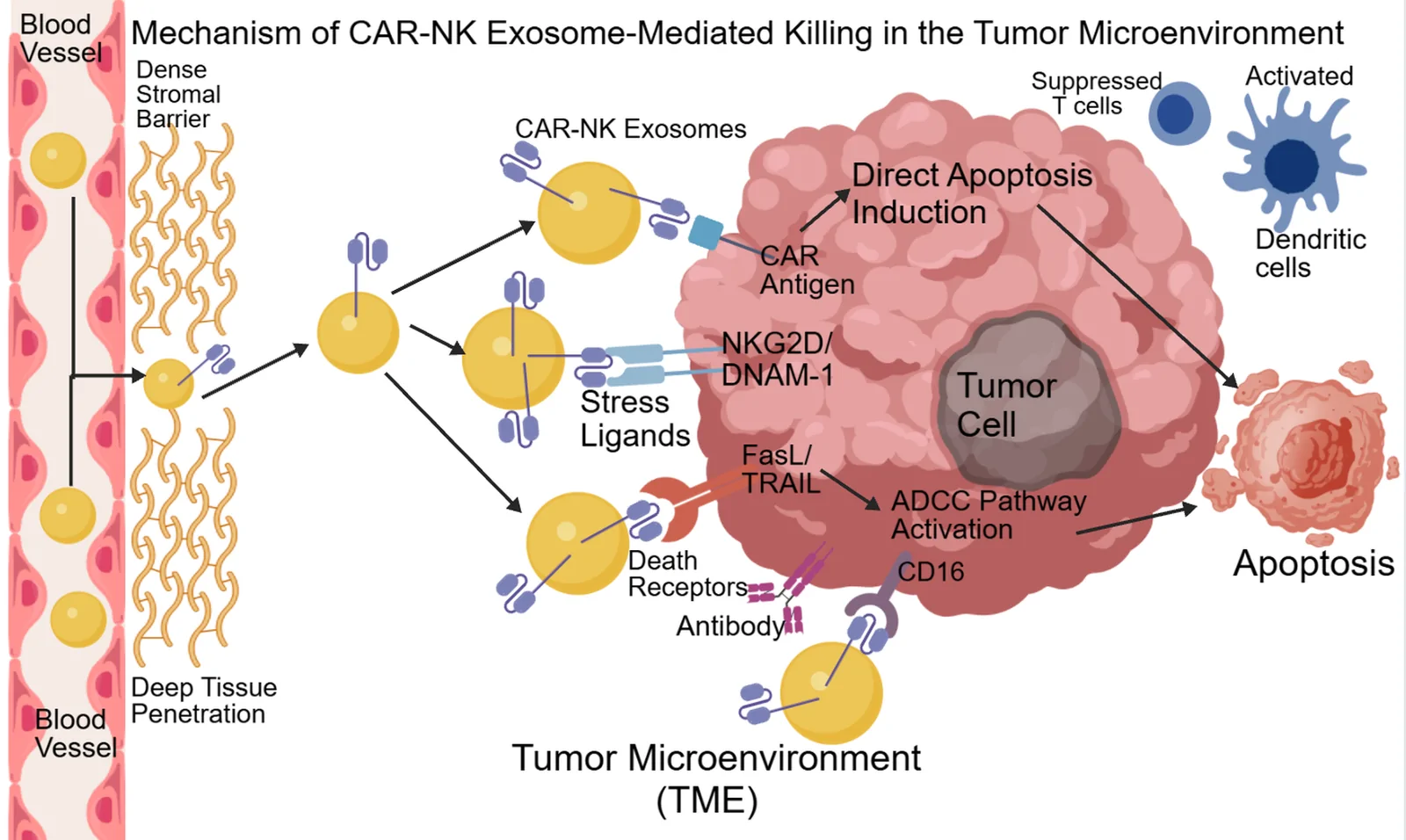
CAR-NK EV/sEV-mediated tumor engagement within the tumor microenvironment. After systemic administration, CAR-NK EVs/sEVs are expected to compete with rapid hepatic and splenic sequestration by the mononuclear phagocyte system before reaching lung tumors. After pulmonary administration, mucus clearance and uptake by airway or alveolar macrophages may similarly reduce the fraction available for tumor-cell engagement. The extent of intact-vesicle tumor exposure, stromal penetration, and retention therefore remains product-, model-, and route-dependent. In the TME, CAR-displaying EVs/sEVs may engage antigen-positive tumor cells through CAR–antigen recognition and additional activating interactions (e.g., NKG2D/DNAM-1 with stress ligands), and can trigger apoptosis via death receptor pathways (FasL/TRAIL). CAR-NK exosomes may also support antibody-dependent mechanisms by interacting with tumor-bound antibodies through CD16, thereby enhancing antibody-dependent tumor engagement/uptake and cytotoxic payload delivery. Collectively, these routes converge on direct apoptotic induction and tumor cell elimination while potentially modulating local immune activity. Created with BioGDP.com.

Despite the potency of engineered CAR-NK cells, their infiltration into solid tumors is physically constrained by the fibrotic extracellular matrix and aberrant vasculature of NSCLC (42). Small EV-enriched preparations are hypothesized to improve access to selected stromal barriers, although this remains context-dependent and requires direct validation in NSCLC models. (1) Potential Access to Selected Tissue Compartments: The smaller size of EV/sEV-enriched preparations may permit access to tissue spaces that exclude intact lymphocytes, but particle size alone does not establish deep or uniform tumor penetration. Reported distribution is influenced by EV source, surface composition, extracellular matrix properties, administration route, tumor architecture, and the labeling or imaging method used (43). (2) Cytotoxic Payload Delivery: Selected CAR-engineered NK-EV preparations have been reported to contain CAR molecules and cytotoxic proteins, but the fraction of CAR-positive vesicles, cargo stoichiometry, and contribution of vesicular versus co-isolated soluble components vary across studies. Antigen-dependent activity should be demonstrated using matched CAR-negative EVs, antigen-negative target cells, antigen-blockade experiments, and dose-normalized comparisons (44). (3) Versatile Cargo Loading: Beyond endogenous proteins, the lipid bilayer of exosomes allows for the encapsulation of exogenous therapeutics. EV/sEV preparations can be engineered to carry therapeutic molecules and may be formulated for nebulization or dry-powder delivery. However, pulmonary delivery should not be inferred from nanoscale size alone. After inhalation, vesicles deposited in conducting airways may be trapped in mucus and removed by mucociliary transport, whereas vesicles reaching distal airspaces may be internalized by alveolar macrophages or retained in non-tumor parenchyma. Formulation stability, regional deposition, intact-vesicle recovery, clearance kinetics, and tumor-to-normal-lung exposure therefore require route-specific validation (45–47) (**Figure 2**).

### 3.4 Cell-free EV/sEV products redistribute, rather than eliminate, safety risks

Safety remains a central consideration for any CAR-directed immunotherapy (48). Compared with living CAR-T or CAR-NK cells, CAR-NK EV/sEV products may reduce selected risks that are specifically linked to viable cell administration, including uncontrolled *in vivo* expansion, long-term persistence, replication-competent cellular contaminants, and expansion-driven cytokine amplification (24, 49). As acellular vesicles, EVs cannot proliferate or engraft, and their dose can be defined by particle number, protein content, cargo content, or CAR-positive vesicle fraction (44, 50). However, these features should not be interpreted as general proof of minimal immunogenicity or predictable pharmacokinetics. EV immunogenicity remains dependent on the producer cell source, surface proteins, engineered ligands, luminal cargo, residual soluble factors, purity, dose, administration route, and host immune status. Similarly, EVs can be dosed using quantitative analytical units, but their pharmacokinetics, biodistribution, clearance, and tumor exposure remain incompletely predictable (51). Following systemic administration, a substantial fraction of EVs may be removed rapidly from the circulation by Kupffer cells, splenic macrophages, and other components of the mononuclear phagocyte system, resulting in predominant liver and spleen exposure rather than tumor-selective delivery. Macrophage depletion slows the disappearance of intravenously administered EVs in animal models, supporting a causal role for phagocytic clearance (52). However, the magnitude and kinetics of this process vary with producer-cell source, EV surface composition, dose, administration route, labeling method, and host immune status. Local pulmonary administration may reduce selected systemic distribution steps but replaces them with mucus entrapment, mucociliary transport, alveolar macrophage uptake, and heterogeneous regional ventilation (53). Therefore, the safety rationale for iPSC-derived CAR-NK EV/sEVs should be framed as risk redistribution rather than risk elimination. Living-cell-associated risks may be reduced, but antigen-dependent on-target/off-tumor toxicity, off-tissue biodistribution, unintended immune activation, and repeat-dose immunogenicity remain product-specific risks that require direct evaluation (54, 55).

## 4 Building a standardized CAR-NK EV production platform

iPSC-derived NK cells can provide a renewable clonal producer-cell source and may reduce donor-to-donor variability. This source-level advantage does not, however, guarantee homogeneous EV populations or reproducible cargo loading, because EV biogenesis remains sensitive to differentiation state, producer-cell activation, culture conditions, harvest timing, and downstream processing. Standardized iPSC differentiation protocols optimized for NK-cell fate may improve producer-cell consistency, although phenotypic and functional comparability must be demonstrated across independent manufacturing runs. Recent reviews indicate that iPSC-NK cells can be engineered to express CARs targeting antigens such as EGFR or HER2, resulting in potent antitumor activity in preclinical models. For example, iPSC-NK cells engineered with an EGFR-specific CAR showed potent antitumor activity in a glioblastoma model (56). The use of iPSC-derived NK cells therefore supports a standardized platform by reducing donor variability and permitting the efficient incorporation of CARs and supportive transgenes, such as IL-15, into a renewable cell source (57).

### 4.1 Scalable producer-cell expansion supports EV/sEV manufacturing

Scalable bioreactor culture is critical for expanding CAR-NK cells to produce high EV yields. Stirred-tank and hollow-fiber bioreactors allow much greater cell density than static flasks. A stirred-bioreactor study reported approximately 1000-fold expansion of iPSC-derived NK cells with comparable cell quality during scale-up from 1 L to 10 L systems. This supports cell-source scalability, but it should not be interpreted as evidence of iPSC-CAR-NK EV yield, EV potency, or clinical EV dose feasibility (58). Closed-system perfusion bioreactors can also continuously harvest EVs: St-Denis-Bissonnette et al. reported a clinically relevant workflow for producing NK cells and NK-derived EVs at large scale, with batch outputs on the order of >10^13 EVs. This study supports the feasibility of large-scale NK-EV biomanufacturing and showed that intravesicular cytotoxic cargo, including perforin and granzymes, could be preserved. However, it does not directly establish the yield, purity, CAR-positive vesicle fraction, or potency of iPSC-derived CAR-NK EV/sEV products (59). Similarly, Kink et al. reported that growing human MSCs in a hollow-fiber bioreactor yielded ~38-fold higher EV harvests by volume compared to planar flask culture (60). Notably, even GMP-like hollow-fiber systems reporting >10^13 EVs per batch illustrate the right order of magnitude, but repeated dosing at 10^12–10^13 EVs per administration could still translate into multiple batches per patient course unless yields and purification throughput are further improved. Collectively, these studies support the scalability of related cell and EV manufacturing systems, but they do not establish process performance for iPSC-CAR-NK EV/sEVs. Platform-specific studies are required to define EV yield, CAR-positive vesicle fraction, cargo composition, downstream recovery, purity, and lot-to-lot potency under matched manufacturing conditions. Closed and automated bioreactors may reduce operator-dependent variability, but scale-up should be evaluated using dose-relevant metrics, including recoverable CAR-positive vesicles or validated potency units per liter per culture day, downstream recovery, number of clinical doses per batch, and lot-failure rate. Total particle yield alone is insufficient because scale-up may increase non-functional vesicles or process-related impurities without proportionally increasing active product.

### 4.2 GMP-compatible isolation and purification define EV/sEV product quality

After expansion, EVs must be isolated and characterized in a standardized workflow. Scalable filtration and chromatography methods (rather than labor-intensive ultracentrifugation) are preferred for GMP production (61). For example, automated platforms often employ tangential flow filtration (TFF) to concentrate and purify EVs, as TFF is both efficient and scalable (62). In practice, the use of TFF, often combined with size-exclusion or affinity-based purification, can substantially improve recovery. Wiest et al. reported that TFF produced an approximately 140-fold higher EV yield than ultracentrifugation in a two-dimensional culture system while enabling sterile, closed-system processing (63). For an iPSC-derived CAR-NK EV/sEV product, release testing should minimally cover identity, purity, safety, and mechanism-linked potency, including EV particle size and concentration, EV-associated markers, CAR-positive vesicle fraction, CAR density, NK-related markers, residual host-cell DNA and proteins, residual cytokines, sterility, endotoxin, mycoplasma, non-vesicular contaminants, and antigen-dependent functional activity.

### 4.3 Batch-to-batch consistency: quality control of EV identity and potency

Beyond total particle numbers, clinical translation requires stringent quality control of the EV product. A clonal iPSC source may reduce one component of variability, namely donor-to-donor differences, but does not remove variability arising from differentiation, activation, EV biogenesis, purification, or formulation (64). Every lot originates from the same clonal iPSC master cell bank, which is expected to reduce donor-to-donor variability. However, EV composition can still vary with producer-cell state and culture conditions (e.g., stimulation, hypoxia, media), so batch comparability must be empirically demonstrated using orthogonal identity and cargo assays. For example, Korchak et al. developed a multiplex bead-based flow assay covering 37 surface markers and demonstrated a correlation coefficient of r ≥ 0.90 among successive EV batches from the same source, whereas batches from different sources did not show comparable correlations (65). In practice, a GMP process would profile each EV lot’s surface (CD9, CD63, CD81, plus any CAR or NK markers) and cargo (e.g. granzyme/TSG101 versus albumin) to ensure sameness (66). Regulatory guidance (e.g. ISEV’s MISEV criteria) requires defining EV purity (absence of cellular debris proteins like calnexin) and quantifying particle size/distribution (67). Although a clonal iPSC master cell bank may reduce donor-derived variability, it does not by itself guarantee stable EV biogenesis, cargo loading, or surface composition. Lot comparability must therefore be demonstrated empirically using orthogonal identity assays, impurity profiling, cargo measurements, CAR-positive EV quantification, and dose-normalized potency testing across multiple manufacturing runs. For CAR-displaying EVs, the fraction of CAR-positive vesicles and CAR density per vesicle should be quantified, rather than assumed, using assays such as bead-based flow cytometry, ELISA, or single-EV imaging. Notably, in a CAR-NK92 model, CAR engineering did not significantly change exosome particle yield or total protein content while enabling CAR incorporation on EVs, suggesting that CAR expression does not necessarily compromise EV output, although this remains construct- and process-dependent. A single marker correlation threshold should not be used as the sole release criterion. Instead, batch release should rely on a matrixed specification that integrates EV identity, impurity limits, CAR-positive vesicle fraction, cargo content, sterility/safety tests, and at least one mechanism-linked potency assay.

### 4.4 Process blueprint: manufacturing workflow and platform design

A generalized manufacturing blueprint for iPSC-derived CAR-NK EVs begins with a master iPSC cell bank that has been clonally selected, karyotyped, and engineered with the desired CAR construct and safety modifications (68). This bank feeds a seed train: iPSCs are expanded in feeder-free suspension (e.g. “spin” embryoid bodies or 2D aggregates) and driven to a hematopoietic NK lineage under defined cytokines (69). Feeder-free “spin EB” protocols yield CD34+ precursors in 6–11 days, then NK differentiation continues for a few weeks. The resulting iPSC-derived NK cells, typically expressing CD56 and CD16, are transferred to a large-scale culture system, such as a stirred-tank or hollow-fiber bioreactor, in which the cells can be maintained for several days or weeks. The medium is serum-free and EV-friendly (often including EV-depleted supplements) and can be implemented using defined, xeno-free ancillary materials. Importantly, many key reagents used for iPSC culture and NK differentiation/activation (including defined media and commonly used cytokines) are available in GMP/clinical-grade formats, which helps de-risk CMC translation. During expansion, conditioned medium is continuously harvested. In a hollow-fiber bioreactor, the medium flows across a semipermeable membrane; the cells are retained within the reactor, whereas EV-containing material can be collected from the appropriate harvest compartment. This “perfusion” harvest can be done daily (70). To increase EV release or alter EV cargo, producer cells may be exposed to activation cytokines, such as IL-15 and IL-18, or to defined stress conditions that mimic memory-like activation; however, the resulting effects on EV identity, purity, and potency require direct validation (71). The collected medium is then clarified (e.g., closed filtration to remove cells and debris), and EVs are concentrated and buffer-exchanged using scalable tangential flow filtration (TFF). Further purification can be performed using size-exclusion chromatography (SEC) and/or other chromatography/filtration steps preferred over ultracentrifugation for GMP-aligned scale-out. Closed-loop perfusion bioreactors (including hollow-fiber systems) enable repeated or continuous EV harvest under sterile, tubing-welded conditions and have been used as scalable platforms for EV manufacturing. Downstream, automation-friendly, closed workflows integrating in-line clarification with tangential flow filtration (TFF) and optional size-exclusion chromatography (SEC) are increasingly used to improve reproducibility and GMP compatibility. Finally, EVs are formulated (e.g. in buffer with cryoprotectant), vialed, and frozen under defined conditions. An iPSC master cell bank can improve source control, but reproducibility must be demonstrated rather than assumed at each unit operation. Differentiation efficiency, producer-cell phenotype, EV yield, CAR display, cargo composition, impurity profile, and potency should be monitored across independent runs and after relevant process changes. Closed and automated systems may reduce operator-dependent variability, but they cannot eliminate biological drift, process-dependent changes in EV composition, or batch failure. Experience with living iPSC-NK products supports the feasibility of clonal cell banking and closed producer-cell workflows, but EV harvest, purification, active-dose definition, formulation, stability, and process comparability constitute a distinct CMC program and should not be regarded as a simple extension of the cellular manufacturing process (23).

### 4.5 Cost of goods, regulatory acceptance, and commercial feasibility

No platform-specific cost-of-goods dataset was identified for iPSC-derived CAR-NK EV/sEV products. Relevant cost drivers are expected to include GMP-grade iPSC maintenance and NK differentiation, cytokines and defined media, bioreactor operation, repeated conditioned-medium harvest, TFF and chromatography consumables, purification-associated product loss, single-EV and cargo analytics, microbiological release testing, fill-finish, storage, transport, and batch rejection (72). A published EV bioprocess economic model identified labor and consumables during EV harvest as major cost drivers, supporting automation and higher-throughput downstream processing; however, cost estimates derived from other producer-cell systems cannot be transferred directly to iPSC-CAR-NK EVs (73). Commercial scalability should be defined by potency-adjusted yield and clinical doses per batch rather than by total particle count. Feasibility will depend on the intended clinical dose, dosing frequency, fraction of CAR-positive vesicles, purification recovery, formulation volume, shelf life, release-test turnaround time, storage losses, and whether pulmonary administration requires a dedicated delivery device. Process intensification is commercially useful only if EV identity, cargo composition, CAR display, purity, and functional potency remain comparable across scale (74). Batch-to-batch variability should be assessed using multiple independent upstream and downstream manufacturing runs rather than technical replicates from a single harvest. Comparability protocols should prospectively define acceptable changes in producer-cell phenotype, EV yield, CAR-positive fraction, cargo distribution, impurity profile, and relative potency after changes in culture scale, raw materials, bioreactor configuration, purification membranes, formulation, storage, or delivery device. A clonal master cell bank reduces donor variability but does not eliminate process-dependent variation in EV biogenesis or cargo loading (75). Regulatory acceptance will require a clearly defined drug substance and drug product, justified critical quality attributes, controlled critical process parameters, qualified or validated analytical methods, stability data, and comparability after manufacturing changes. FDA has stated that exosome products intended to treat human disease are regulated as drugs and biological products and require premarket review and approval. Early interaction with the relevant regulatory authority is therefore warranted, particularly for genetically engineered EVs and inhaled formulations. Commercial feasibility should be presented as an unresolved development question rather than as an inherent advantage of the platform (76).

### 4.6 Regulatory classification and GMP expectations in the United States and European Union

In the United States, FDA states that exosome products intended to diagnose, treat, mitigate, or prevent human disease are generally regulated as drugs and biological products under the Federal Food, Drug, and Cosmetic Act and the Public Health Service Act. Clinical investigation therefore requires an effective investigational new drug application, and marketing requires premarket review and approval. FDA continues to state that no exosome product has received FDA approval. Manufacturing must follow phase-appropriate current good manufacturing practice and product-specific chemistry, manufacturing, and controls requirements, including controls for starting materials, identity, purity, potency, sterility, endotoxin, process-related impurities, stability, and manufacturing comparability. FDA has not established a dedicated, universally applicable EV release standard. Its regulatory-science activities explicitly identify the absence of standardized EV manufacturing quality-control methods as an unresolved problem (77, 78). Consequently, compliance with generic EV markers or MISEV reporting recommendations would not be sufficient for an IND; each product would require a justified definition of the active substance, critical quality attributes, mechanism-linked potency, dose unit, and manufacturing controls. In the European Union, EV-based therapeutics do not have a single dedicated legal category, and regulatory classification should be determined case by case according to the final product, active components, manufacturing process, and mechanism of action. Derivation from genetically engineered iPSC-CAR-NK producer cells should not by itself be treated as proof that the final acellular product is an advanced therapy medicinal product. Developers should seek early advice from the relevant national competent authority and, where ATMP status is uncertain, a scientific classification recommendation from EMA’s Committee for Advanced Therapies. If the final EV product is regulated as a non-ATMP biological medicinal product, the applicable EU framework would include the general EudraLex Volume 4 requirements, sterile-product controls in Annex 1, and biological-product provisions in Annex 2. If the product is classified as an ATMP because of its composition or mechanism of action, the ATMP-specific GMP requirements in EudraLex Volume 4 Part IV and the corresponding EMA quality, nonclinical, and clinical guidance would apply. An EV product that delivers a recombinant nucleic-acid sequence through a gene-therapy mechanism may require a different classification from an EV whose principal activity is mediated by surface CAR binding and preformed cytotoxic proteins. Regardless of classification, the EU dossier would be expected to define the producer-cell bank, raw-material controls, manufacturing process, drug substance and drug product, specifications, impurities, stability, and comparability (44, 77). The current EMA ATMP guideline also emphasizes characterization of iPSC-related starting materials and expects medicinal-product specifications to include identity, content, purity, sterility and endotoxin where applicable, together with a potency test unless omission is scientifically justified. ISEV should be distinguished from FDA and EMA because it is a scientific society rather than a regulatory authority. MISEV2023 provides consensus recommendations for EV nomenclature, source and culture reporting, separation, characterization, functional studies, and *in vivo* experiments. These recommendations improve scientific rigor and can inform product development, but they do not define legal classification, confer GMP compliance, establish regulatory release specifications, or replace FDA or EU requirements. ISEV position papers and its Regulatory Affairs and Clinical Use Task Force support early definition of the intended product, traceable producer-cell sources, rigorous EV and non-EV characterization, mechanism-linked functional testing, safety evaluation, and application of the existing regulatory framework in each jurisdiction. Thus, the manuscript should refer to an ‘ISEV scientific-consensus perspective’ rather than an ‘ISEV regulatory requirement’ (79, 80).

### 4.7 Minimum release testing, interlaboratory standardization, and potency bridging for CAR-NK EV/sEV products

Clinical translation of iPSC-derived CAR-NK EV/sEV products requires a predefined release panel that goes beyond generic EV characterization (81, 82). In accordance with MISEV2018 and EV-TRACK principles, the manufacturing process should report the producer-cell source, culture conditions, separation and purification methods, particle measurements, EV-associated and negative markers, impurity profile, and functional assays (79, 83). For a CAR-NK EV/sEV product, release testing should be organized around four categories: identity, purity, safety, and potency (84). Identity testing should confirm that the final product is an EV/sEV-enriched preparation derived from the intended iPSC-CAR-NK producer cells. Minimum identity attributes should include particle size distribution, particle concentration, EV-associated markers such as CD9, CD63, CD81, TSG101 or ALIX, NK-related markers where relevant, producer-cell identity markers, CAR-positive EV fraction, and CAR density per EV or per particle-equivalent dose (85). Because CAR incorporation into EVs can vary with construct design, producer-cell activation state, culture conditions, and purification strategy, CAR display should be quantified rather than assumed (24). Purity and safety testing should define both vesicular and non-vesicular components (86). Minimum purity attributes should include residual host-cell DNA, residual host-cell proteins, albumin or lipoprotein contamination, residual cytokines such as IL-15, residual free antibody or free CAR-binding ligand, residual vector- or process-related impurities where applicable, and depletion of cellular debris markers such as calnexin (86). Microbiological release testing should include sterility, endotoxin, mycoplasma, and adventitious-agent risk control according to the intended clinical product class and manufacturing process (87). Potency testing should quantify a biological activity attributable to the proposed mechanism of action and should not be inferred from particle concentration, EV-marker abundance, CAR abundance, or cytotoxic-cargo content alone. For a CAR-NK EV/sEV product, the primary cell-based assay should measure antigen-dependent cytotoxicity using matched target cells that differ only in target-antigen expression. The assay should include CAR-negative EVs generated from the same parental producer-cell background, antigen-blocking or competition controls, EV-depleted process controls, and dose-response testing expressed in both total particles and CAR-positive vesicles or another justified potency-linked dose unit. Cross-laboratory standardization requires control of the target-cell clone, passage range, antigen density, target-cell density, culture medium, serum or protein matrix, EV thawing and mixing procedure, incubation time, readout platform, and statistical analysis model (24, 44). Each run should include qualified positive and negative reference lots and predefined system-suitability criteria. Relative potency should be estimated against an internal reference standard using parallel dose-response curves, and the assay should be evaluated for specificity, accuracy, precision, linearity, range, robustness, and stability-indicating capacity. Interlaboratory transfer studies and shared reference materials are required before numerical potency values can be compared across manufacturing or testing sites. Analytical-reporting frameworks such as MISEV2023 and MIFlowCyt-EV can improve reproducibility of EV characterization, single-EV flow-cytometry measurements, and reporting of experimental variables, but they do not by themselves establish a standardized functional potency assay. Interlaboratory studies have shown that differences in EV isolation and analytical workflows can materially alter the resulting preparation and measured attributes. Assay transfer must therefore include both the EV test article and the complete procedure for sample preparation, instrument calibration, data acquisition, normalization, and analysis (88). Currently available *in vitro* assays are not sufficiently standardized or clinically validated to predict the efficacy of iPSC-derived CAR-NK EV/sEVs in NSCLC. Antigen binding, cellular uptake, and short-term cytotoxicity may support lot release and manufacturing comparability, but they do not capture pulmonary deposition, mucus clearance, macrophage sequestration, stromal access, antigen heterogeneity, or repeat-dose immune effects. Clinical predictiveness should therefore be developed by correlating release assays with orthogonal three-dimensional tumor models, *in vivo* tumor exposure and pharmacodynamic endpoints, and eventually patient pharmacokinetic, pharmacodynamic, and response data. Until such correlations are established, potency assays should be described as mechanism-linked quality-control tools rather than validated surrogate endpoints for clinical efficacy (44). A matrixed potency strategy should therefore combine: (i) antigen-specific binding or uptake; (ii) antigen-dependent cytotoxicity; (iii) CAR-positive vesicle fraction and CAR density; and (iv) one or more mechanism-relevant cargo or immune-modulation assays. The primary release assay should be complemented by orthogonal methods, but multiple weakly informative assays should not be treated as equivalent to one validated mechanism-linked potency assay. Acceptance ranges should initially support manufacturing consistency and should only be interpreted as efficacy-predictive after prospective nonclinical and clinical bridging (**Table 2**).

**Table 2:** Proposed minimum release panel for iPSC-derived CAR-NK EV/sEV products.

Release domain	Minimum attribute	Example assay or readout	Rationale
Identity	Particle size distribution	NTA, TRPS, DLS, TEM or cryo-EM	Confirms sEV-enriched particle profile and detects aggregation
Identity	Particle concentration	NTA, TRPS, high-sensitivity flow or orthogonal particle counting	Defines dose input and lot-to-lot particle consistency
Identity	EV-associated markers	CD9, CD63, CD81, TSG101, ALIX by immunoblot, ELISA, bead-based flow or single-EV analysis	Confirms EV/sEV enrichment
Identity	NK-related markers	CD56, CD16, NKG2D, DNAM-1 or other product-relevant markers	Confirms source-cell-related EV features where incorporated
Identity	CAR-positive EV fraction	Bead-based flow cytometry, ELISA, antigen-capture assay, single-EV imaging	Quantifies the fraction of EVs carrying the targeting module
Identity	CAR density	CAR signal per particle-equivalent dose, antigen-binding units, single-EV or calibrated bead assay	Supports dose normalization and lot comparability
Identity	Producer-cell identity	iPSC-CAR-NK cell phenotype, CAR copy/insertion characterization, NK differentiation markers	Links final EV product to the intended producer-cell platform
Purity	Residual host-cell DNA	qPCR, PicoGreen, ddPCR	Controls producer-cell and process-related nucleic acid impurities
Purity	Residual host-cell proteins	Total protein profile, host-cell protein assay, proteomics	Detects non-vesicular protein contamination
Purity	Albumin/lipoprotein contamination	Albumin, ApoA1, ApoB or lipoprotein particle assays	Distinguishes EVs from common extracellular contaminants
Purity	Cellular debris markers	Calnexin, GM130, cytochrome c or other depleted markers	Demonstrates depletion of intracellular organelle or cell-fragment contaminants
Purity	Residual cytokines	Free IL-15, IL-2, IL-12, IL-18 or process-specific cytokines	Controls carryover that could cause unintended immune activation
Purity	Residual free targeting ligand	Free antibody, soluble CAR-binding ligand, free scFv or Fc-binding reagent	Distinguishes EV-associated targeting from soluble ligand carryover
Safety	Sterility	Compendial sterility test or validated rapid microbiological method	Required microbiological safety attribute
Safety	Endotoxin	LAL or recombinant factor C assay	Controls innate immune activation risk
Safety	Mycoplasma	Culture, PCR or validated rapid test	Required safety testing for cell-derived products
Safety	Adventitious-agent risk	Process-specific viral and adventitious-agent testing	Addresses producer-cell and culture-material risks
Potency	Antigen-specific binding or uptake	Antigen-positive versus antigen-negative NSCLC cells; quantitative uptake imaging or flow assay	Demonstrates target-dependent engagement
Potency	Dose-response cytotoxicity	Viability, apoptosis, impedance or live-cell imaging assay across EV dose range	Defines functional activity and dose response
Potency	CAR-dependence	Antigen blockade, soluble antigen competition, CAR-negative EV control	Confirms that activity depends on CAR-mediated targeting
Potency	Cytotoxic cargo	Granzyme B, perforin, FasL, TRAIL by ELISA, immunoblot or proteomics	Supports mechanism-linked cargo comparability
Potency	TME modulation, if claimed	Macrophage polarization, dendritic-cell activation, T-cell recruitment, TGF-β pathway readout	Required only if immunomodulatory claims are made
Potency bridging	In vivo or surrogate bridge	Tumor-control model, biodistribution-linked exposure assay, validated in vitro surrogate	Tests whether in vitro potency predicts in vivo activity
Comparability	Matrixed lot analysis	Particle number, CAR-positive fraction, cargo content, killing activity, impurity profile across lots	Establishes batch consistency and release acceptance ranges

The table lists proposed release attributes for identity, purity, safety, potency, and potency bridging. This panel is intended as a translational framework rather than a finalized regulatory specification. Assays and acceptance criteria should be product-specific and justified by the proposed mechanism of action, manufacturing process, route of administration, and nonclinical validation data. EV terminology and reporting should follow MISEV2018 and EV-TRACK principles, including transparent reporting of producer-cell source, culture conditions, isolation and purification methods, EV-associated markers, negative or depleted markers, impurities, and functional assays.

### 4.8 Preclinical prototypes and transition toward clinical development

A limited number of proof-of-concept studies support the feasibility of displaying CAR molecules on EVs and achieving antigen-associated activity in selected non-iPSC models. The evidence remains constrained by small study numbers, heterogeneous producer cells, variable EV characterization, inconsistent dose units, and the absence of direct testing of iPSC-derived CAR-NK EV/sEVs in NSCLC (24). For example, Tîrziu et al. isolated EVs/sEVs described by the original authors as exosomes. These HER2-targeted CAR EVs displayed CAR proteins on their surface and selectively killed HER2-positive tumor cells *in vitro*, supporting the possibility that CAR surface display can confer antigen-specific binding or uptake and facilitate cytotoxic cargo delivery (89). Notably, canonical intracellular CAR signaling (e.g., CD3ζ ITAMs, CD28/4-1BB) is not expected to operate within EVs due to the lack of a complete signaling apparatus; thus, the CAR functions primarily as a targeting ligand on the EV surface. In another study, EGFR-targeted CAR-NK EVs showed substantially greater tumor control than unmodified NK-cell-derived EVs *in vivo*. These findings establish mechanistic plausibility for CAR-displaying NK-EVs in another solid-tumor context, but they cannot be assumed to predict antigen coverage, biodistribution, stromal access, or therapeutic efficacy in NSCLC. EGFR-directed CAR-NK EVs therefore require direct testing in antigen-heterogeneous lung-cancer models and comparison with EGFR-negative normal tissues. Beyond CAR targeting, other engineering has shown promise. Memory-like NK cells preactivated with IL-12, IL-15, and IL-18 have been reported to produce EVs enriched in granulysin and granzymes, with enhanced cytotoxic activity against lung cancer cells (43). Likewise, EVs from IL-15/IL-21 stimulated NK92 cells showed enhanced cytotoxic markers and could be further loaded with drugs. Cytokine priming has altered EV cargo and activity in selected non-iPSC NK systems; however, whether the same intervention reproducibly improves iPSC-CAR-NK EV potency without increasing cargo heterogeneity, residual-cytokine contamination, or unintended immune activation remains unknown (90). While these are not yet CAR EVs per se, they exemplify how defined iPSC-derived NK can be manipulated to tune the EV content. In the published phase 1 FT596 trial, 86 patients with relapsed or refractory B-cell lymphoma received iPSC-derived CD19 CAR-NK cells with or without rituximab. The RP2D was preliminarily identified as 1.8 × 10^9 cells administered as three doses per cycle. CRS was reported in 1/18 patients in regimen A and 9/68 patients in regimen B, with no observed neurotoxicity. These data support the clinical feasibility of iPSC-derived CAR-NK cell manufacturing and dosing, but they do not establish efficacy, dosing, or safety for iPSC-derived CAR-NK EVs/sEVs in NSCLC (91).

## 5 Comprehensive engineering strategies: from genetic code to nanocarrier

**Figure 3:**
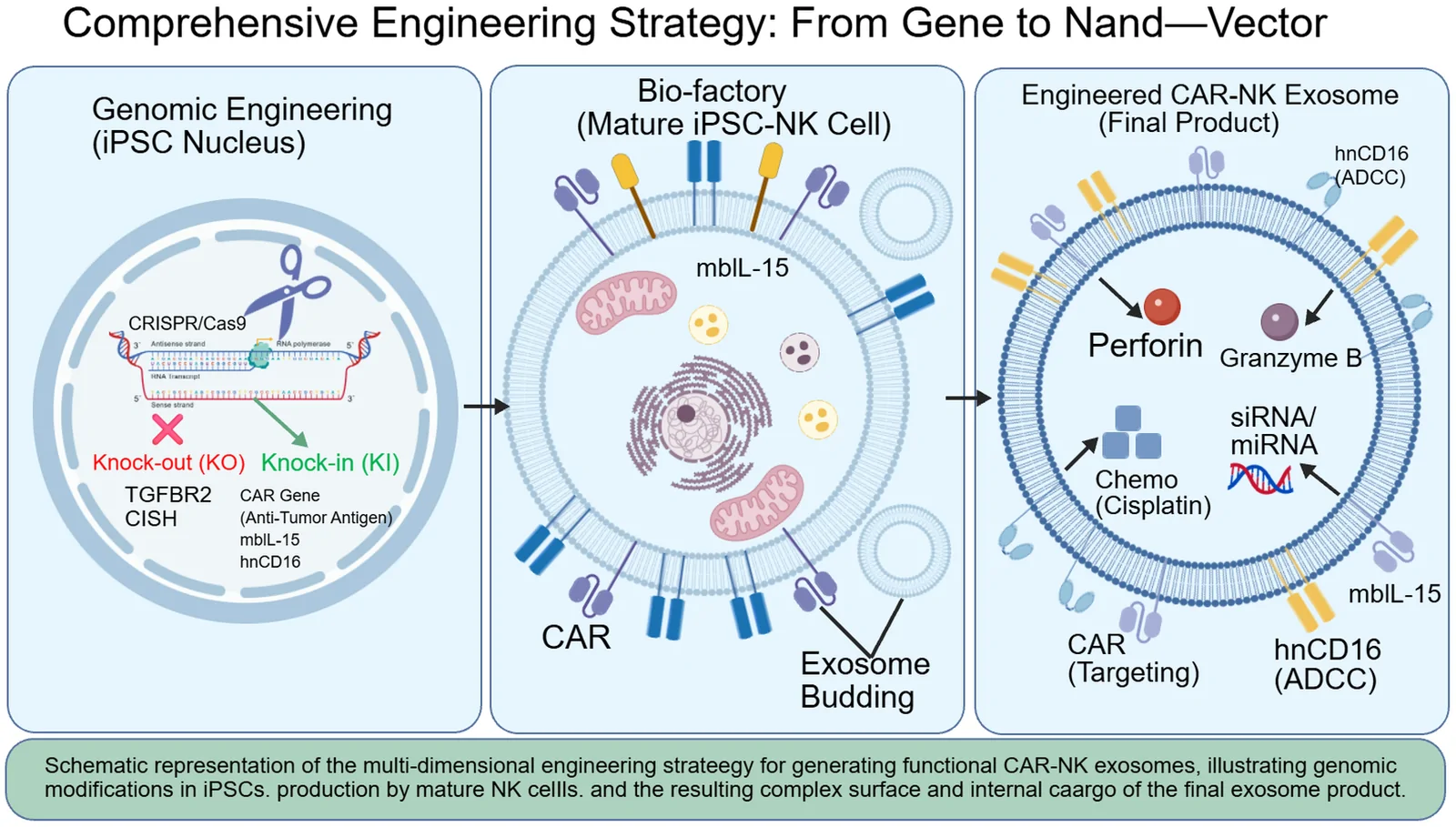
Comprehensive engineering strategy from gene editing to multifunctional CAR-NK EVs/sEVs. Schematic of a multi-layer design pipeline for generating engineered CAR-NK exosomes. Genomic engineering (iPSC nucleus): CRISPR/Cas9 enables targeted knock-out (e.g., *TGFBR2*, *CISH*) and knock-in of functional modules (e.g., CAR, mbIL-15, hnCD16). Bio-factory stage (mature iPSC-NK cells): engineered iPSC-derived NK cells stably express surface receptors and cytokine support, and continuously release exosomes via vesicle budding. Final product: CAR-NK EV/sEV-enriched preparations are expected to contain targeting and ADCC-enhancing molecules (CAR and hnCD16) and can be equipped with additional payloads (perforin/granzyme B, regulatory siRNA/miRNA, or chemotherapeutics such as cisplatin), enabling programmable antitumor activity. Created with BioGDP.com.

The differentiation of iPSCs into NK cells provides a unique opportunity to employ a “bottom-up” engineering approach. Unlike primary NK cells, which are refractory to genetic manipulation, iPSCs serve as a stable molecular chassis that can be extensively edited to introduce multiple functionalities. This section outlines a multi-layered engineering strategy—ranging from upstream genomic editing of the producer cells to downstream surface and luminal modification of their secreted exosomes—designed to overcome the specific barriers of non-small cell lung cancer (NSCLC) (92) (**Figure 3**) (**Tables 3**–**5**).

**Table 3:** Representative evidence supporting CAR-, NK-, and EV-based engineering strategies relevant to iPSC-derived CAR-NK EV/sEV development.

Source cell	Engineered target / EV design	EV terminology used in source	Model	Key outcome	EV characterization performed	Potency assay	Main limitations	Primary citation
CAR-T cells	CAR-displaying EVs; antigen-specific CAR transferred from effector CAR-T cells	"CAR exosomes"	Preclinical tumor models; mainly non-NSCLC solid-tumor models	CAR-containing EVs showed antigen-dependent antitumor activity and lower cytokine-release signals than parental CAR-T cells in preclinical testing	Particle analysis and EV marker assessment reported; CAR expression on EVs evaluated	Antigen-specific tumor-cell killing; in vivo tumor growth inhibition; cytokine-release comparison	Not NSCLC-focused; T-cell rather than NK-cell source; "exosome" terminology may not prove endosomal origin; clinical dose and PK unknown	Fu et al., 2019 (24)
CAR-T cells	Mesothelin-targeted CAR-T-derived EVs	"Exosomes"	MSLN-positive triple-negative breast cancer: in vitro and mouse models	MSLN-CAR EVs targeted MSLN-positive tumor cells and reduced tumor growth	EV morphology, particle-size assessment, EV markers, and CAR-related characterization reported	MSLN-dependent uptake/killing; tumor-growth inhibition	Breast cancer, not lung cancer; CAR-T rather than CAR-NK source; EV biodistribution and repeat-dose safety remain limited	Yang et al., 2021 (93)
CAR-T cells	Mesothelin-targeted CAR-T EVs loaded with paclitaxel for inhalation	"CAR-T cell-derived exosomes"	Lung cancer model; inhalation delivery	Inhaled PTX@CAR-Exos accumulated in tumor regions, reduced tumor burden, prolonged survival, and altered the immune microenvironment in preclinical lung cancer models	Particle-size distribution, morphology, EV markers, drug loading, and CAR-related features reported	Tumor targeting, cytotoxicity, in vivo tumor suppression, survival, and immune-microenvironment readouts	CAR-T rather than CAR-NK source; drug-loaded combination product; aerosol stability and human pulmonary deposition remain unresolved	Zheng et al., 2023 (94)
CAR-T cells	CD19-positive CAR-T-derived EVs	"Extracellular vesicles"	CD19-positive leukemia models; patient monitoring and in vitro functional testing	CD19.CAR-positive EVs expressed CAR and contributed to direct antileukemic activity	Circulating CAR-positive EV detection and EV marker characterization reported	CD19-positive leukemia-cell killing	Hematologic malignancy, not NSCLC; mainly biomarker/functional evidence; not an iPSC or NK-derived platform	Lanuti et al., 2025 (89)
CAR-NK cells	HER2-CAR-NK-derived EVs combined with T7-modified micelles / "nano-bomb" system	"CAR-NK cell-derived exosomes"	HER2-positive breast cancer brain metastasis; orthotopic mouse model	Engineered CAR-NK EV-based system improved HER2-positive tumor targeting, crossed the blood-brain barrier in the model, enhanced antitumor activity, and prolonged survival	EV morphology, particle size, EV markers, CAR-related characterization, and cargo formulation reported	HER2-positive tumor-cell killing; biodistribution; survival and tumor-burden assays	Breast cancer brain metastasis, not NSCLC; complex hybrid nanocarrier rather than simple CAR-NK EV product; source was not iPSC-derived	Tao et al., 2023 (22)
CAR-NK92 cells	Anti-HER2 CAR-NK92-derived EVs	"Exosomes"	HER2-positive tumor-cell lines; in vitro	CAR-NK92-derived vesicles contained CAR-related features and showed selective cytotoxicity against HER2-positive tumor cells	Exosome/EV isolation and characterization reported, including particle and marker analyses	HER2-dependent cytotoxicity assays	In vitro only; NK-92 cell line rather than primary or iPSC-NK source; not lung cancer; endosomal origin may not be fully proven	Tirziu et al., 2025 (95)
NK-92 cells	IL-15 and cetuximab-tethered engineered NK-sEVs targeting EGFR	"Small extracellular vesicles"	EGFR-positive lung cancer cells and mouse lung cancer models	Surface-engineered NK-sEVs showed enhanced EGFR-positive tumor binding, direct killing, immune-cell-mediated cytotoxicity, and improved antitumor effects compared with control EVs	sEV particle analysis, marker characterization, and surface-engineering validation reported	EGFR-dependent binding/uptake, apoptosis/cytotoxicity, in vivo tumor suppression	Not a CAR construct; NK-92 rather than iPSC-NK source; surface antibody tethering may not fully model CAR-NK EV biology	Kang et al., 2025 (96)
Primary NK cells	Unmodified or cytokine-activated NK-EVs carrying cytotoxic proteins	"NK-cell-derived EVs/exosomes"	Cancer-cell killing models; some lung-cancer and organoid-related systems	Primary NK-EVs carried cytotoxic cargo and induced tumor-cell killing or immune modulation	EV size, markers, cargo, and source-cell information variably reported	Tumor-cell viability/killing; immune-cell recruitment or modulation	Not CAR-engineered; donor variability; EV characterization varies; indirect comparator evidence only	Wu et al., 2019 (27); Jong et al., 2017 (97); Palade et al., 2025 (98)
iPSC-NK cells	iPSC-derived NK cells as potential producer cells for future CAR-NK EVs	EV product not directly tested	iPSC-NK cell preclinical and clinical-analog studies; no direct EV studies	iPSC-NK cells support clonal manufacturing, genetic engineering, and antitumor activity; these data support producer-cell feasibility but not EV efficacy	Cell-product characterization rather than EV-product characterization	Cell cytotoxicity, persistence, clinical safety or response endpoints	No direct iPSC-derived CAR-NK EV/sEV study in NSCLC; EV cargo transfer, CAR-positive EV fraction, potency, dose, PK, and safety remain unvalidated	Cichocki et al., 2020 (99); Ghobadi et al., 2025 (23)
iPSC-CAR-NK cells	Proposed iPSC-derived CAR-NK EV/sEV product	Proposed terminology: EVs/sEVs	No direct published NSCLC model identified	No direct efficacy, biodistribution, inhalation, or clinical safety evidence currently available	Not applicable	Not applicable	Central evidence gap: all claims should be framed as translational hypotheses requiring EV-level validation	Evidence gap identified in this review

EV terminology follows MISEV2018. Products described as “exosomes” in the original literature are classified here as EVs or sEVs unless endosomal origin was experimentally demonstrated. Evidence from CAR-T EVs, NK-92 EVs, primary NK-EVs, and iPSC-NK cell therapies is considered indirect or clinical-analog evidence unless the study directly tested iPSC-derived CAR-NK EVs/sEVs in NSCLC. EV, extracellular vesicle; sEV, small extracellular vesicle; CAR, chimeric antigen receptor; NK, natural killer cell; NSCLC, non-small cell lung cancer; MSLN, mesothelin; EGFR, epidermal growth factor receptor; HER2, human epidermal growth factor receptor 2.

**Table 4:** Direct engineering strategies expected to be displayed on, or packaged within, iPSC-NK–derived extracellular vesicles (EVs).

Engineering Strategy (Producer-cell / EV)	What changes at the EV level	EV-specific advantage (measurable endpoints)	Required EV-level validation (examples)
Membrane-anchored IL-15 (± IL-15Rα complex)	Potential EV surface display of IL-15/IL-15Rα; and/or producer-cell priming effects reflected in EV cargo	Local immune stimulation (recipient NK/CD8 activation) and/or enhanced EV potency via enriched cytotoxic cargo; not "EV self-persistence"	Quantify EV-associated IL-15/IL-15Rα vs soluble fraction; STAT5 signaling / NK or CD8 proliferation assays; dose-normalized tumor killing assays
High-affinity hnCD16 (non-cleavable, high Fc affinity)	Potential Fc-binding capacity on EV surface	Antibody-guided tethering/retention on opsonized tumor cells (enhanced local EV concentration/uptake); not classical cellular ADCC	Fc-binding assay; uptake on antibody-opsonized targets (± cetuximab); synergy assays vs controls
NKG2D overexpression	Increased density of activating receptor on EV membrane (if incorporated)	Broader tumor engagement (stress-ligand recognition) and reduced antigen-escape risk via an additional binding axis	Ligand-dependent binding/uptake; activity in CAR-antigen low/negative settings; surface quantification of NKG2D on EVs
Cytotoxic cargo loading (e.g., Granzyme B, TRAIL/FasL enrichment)	Increased pro-apoptotic protein content in EV lumen/membrane	Direct, cargo-mediated killing (apoptosis) independent of recipient immune cell proliferation	Proteomics/ELISA for key effectors (GZMB/perforin/TRAIL/FasL); apoptosis assays (caspase, Annexin V); potency unit definition and lot-to-lot comparability
miRNA loading (e.g., miR-186)	Enrichment of defined miRNAs in EV cargo	Pathway-linked TME/tumor modulation (e.g., inhibiting immune-escape signaling and tumor programs)	Confirm miRNA enrichment (qPCR/NGS); target knockdown/pathway readouts; functional rescue under TME-mimicking conditions
(Add to address “persistence” properly) EV exposure/retention engineering (e.g., CD47 display)	Reduced phagocytic clearance / improved in vivo retention	Prolonged effective exposure and improved tumor accumulation/retention without implying EV replication	Biodistribution/PK; macrophage uptake assays; tumor retention imaging; safety cytokine panels

This table summarizes modifications most likely to be physically present at the EV level (e.g., membrane display modules and defined cargo enrichment) and therefore directly relevant to EV function *in vivo*. Reported or hypothesized advantages are stated in EV-specific terms, including tumor engagement/uptake, cargo-mediated cytotoxicity, local immune stimulation in the tumor microenvironment (TME), and improved exposure/retention. For each strategy, recommended EV-level validation assays (identity of the engineered feature on/in EVs, dose-normalized potency, and mechanistic readouts) are listed to support translation and comparability.

**Table 5:** Producer-cell genetic edits that may indirectly tune iPSC-NK EV yield, composition, and potency (requires EV-level confirmation).

Engineering Strategy (Producer-cell)	Why it may matter for EVs (indirect)	EV-specific advantage (hypothesis → test)	Required EV-level validation (examples)
TGFBR2 knockout	Preserves producer-cell function under TGF-β exposure; may maintain EV yield/cargo during conditioning; EVs themselves are not “suppressed” via TGF-β receptor signaling	Manufacturing robustness/consistency and maintained EV potency if production is challenged with suppressive cues	Compare EV yield/cargo/potency ± TGF-β during production; EV multi-omics; functional assays normalized by particle number
CISH knockout	Enhances IL-15 signaling in producer cells; may alter EV biogenesis and cytotoxic cargo loading	Higher EV productivity and/or enriched cytotoxic cargo through producer-state tuning	EV yield per cell; cargo quantification (GZMB/perforin); comparability (proteomics/miRNA); potency assays with defined release criteria

This table lists upstream modifications that are primarily established for NK-cell therapy (e.g., resistance to suppressive cues) but may influence EV products indirectly by altering the producer cell’s activation state, vesicle biogenesis, and cargo loading. Because EVs are non-living and do not execute canonical “cell persistence” programs, the anticipated benefits are framed as manufacturing robustness and/or EV cargo/potency shifts, and each entry is explicitly presented as a testable hypothesis requiring EV profiling (multi-omics/cargo quantification) and functional potency assays under relevant conditions.

EVs are non-living and do not proliferate. Accordingly, terms such as “persistence” refer to EV exposure/retention/biodistribution and/or localized stimulation of recipient immune cells (if cytokine complexes are EV-associated), rather than survival/expansion of the producer NK cells. Strategies in **Table 5** are expected to affect EVs indirectly and must be validated through EV-level characterization and dose-normalized functional testing.

### 5.1 Upstream gene editing builds CAR-NK producer cells with defined functional modules

The primary layer of engineering occurs at the iPSC stage, where stable genetic modifications are introduced to ensure that all differentiated NK “producer” cells inherit specific anti-tumor attributes. For exosomes/EVs, membrane-anchored modules (e.g., CAR, hnCD16) are more likely to be displayed on the EV surface, whereas intracellular pathway edits (and some cytokine-support modules) may influence EV yield and cargo indirectly via changes in producer-cell state and vesicle biogenesis; therefore, EV incorporation and bioactivity should be empirically verified for each construct. (1) Chimeric antigen receptor (CAR) integration: To redirect NK specificity against lung tumors, iPSCs have been engineered to express CARs targeting antigens such as Mesothelin (MSLN) or EGFR. For instance, Jiang et al. demonstrated that iPSC-derived NK cells expressing an MSLN-CAR exhibited robust cytotoxicity against solid tumor lines, with the CAR protein being trafficked effectively to the cell surface (92). These transmembrane CAR proteins have been reported to be displayed on EV membranes in multiple CAR-EV studies and are therefore expected to be present on CAR-NK-derived EVs. In practice, CAR surface density should be quantified as part of EV release testing, because the extent of EV display can vary with construct design and producer-cell state. However, because genetic engineering and the producer-cell activation state can influence EV biogenesis and cargo packaging, CAR-positive EVs should be directly benchmarked against CAR-negative EVs from the same parental line for (i) particle yield and size distribution, (ii) CAR-positive fraction and CAR density per vesicle, and (iii) protein/RNA cargo (e.g., proteomics and small-RNA profiling), alongside matched functional potency assays (20, 100, 101). (2) Enhancing factory fitness and EV potency with IL-15: While IL-15/IL-15Rα engineering was originally developed to improve persistence of infused NK cells, it may also be relevant in an EV-based strategy for two reasons. Because the proposed therapeutic is the EV product rather than the engineered cells, we view mbIL-15/IL-15Rα primarily as a manufacturing and EV-potency module, not as a persistence mechanism for an infused cellular product. First, membrane-bound IL-15/IL-15Rα can support cytokine-independent fitness of the iPSC-NK “producer” cells, which may help stabilize EV yield and consistency during manufacturing. Second, sustained IL-15 signaling can modulate producer-cell activation programs that are known to reshape EV cargo and functional potency. Whether IL-15/IL-15Rα is incorporated into purified EVs and remains bioactive requires direct verification, for example, through quantification of EV-associated IL-15/IL-15Rα after purification and recipient-cell bioactivity assays. Importantly, EV-associated IL-15/IL-15Rα should be distinguished from soluble cytokine carryover, and residual free IL-15 should be controlled as a release attribute; if present, EV-associated IL-15/IL-15Rα could potentially provide localized immune stimulation (trans-presentation) within the tumor microenvironment. This potential benefit should be balanced with safety evaluation, including bystander immune-cell activation readouts and systemic cytokine monitoring *in vivo*. (3) Optimizing ADCC (hnCD16): Standard CD16 receptors on NK cells are prone to shedding by ADAM17 proteases upon activation. By editing iPSCs to express a high-affinity, non-cleavable CD16 (hnCD16) variant, researchers have generated NK cells—and potentially EVs—that maintain stable Fc-mediated antibody binding (e.g., cetuximab). For a cell-free EV product, the expected benefit is improved antibody-guided targeting/uptake and enhanced delivery of EV cytotoxic cargo to antigen-positive tumor cells, rather than classical cellular ADCC. As with other surface modules, hnCD16 display on EVs should be confirmed and quantified during product characterization (20, 102).

### 5.2 Overcoming TME suppression: producer-cell engineering to stabilize EV yield and cargo

Because the proposed therapeutic modality is an acellular EV product, ‘TME suppression’ in this section refers primarily to how suppressive cues can reprogram the *producer* iPSC-NK cells and thereby alter EV yield and cargo, rather than a direct ‘exhaustion’ process acting on EVs. EVs do not proliferate or execute intracellular checkpoint signaling; therefore, these edits are best viewed as upstream levers to stabilize manufacturing and bias EV composition toward a more cytotoxic/immunostimulatory profile. (1) TGF-β receptor knockout: TGF-β is abundant in the NSCLC stroma and suppresses NK-cell metabolism and receptor expression. Thangaraj et al. demonstrated that knocking out the *TGFBR2* gene in iPSC-NK cells prevented TGF-β-induced dysfunction, preserving cytotoxicity even in a TGF-β-rich milieu (103). For an EV-only strategy, the expected benefit is indirect—maintaining cytotoxic producer-cell state under suppressive or stress-mimicking conditions so that released EVs remain enriched for lytic effectors; whether TGFBR2 editing measurably improves EV yield/cargo/potency should be confirmed empirically. (2) Metabolic reprogramming (CISH deletion): Deletion of the *CISH* gene (encoding the cytokine checkpoint CIS) removes an intracellular brake on IL-15 signaling. CISH-knockout iPSC-NK cells exhibit enhanced IL-15 responsiveness and metabolic fitness (e.g. increased mTOR signaling and glycolysis). In an EV manufacturing context, improved producer-cell fitness may support more stable EV biogenesis and more consistent packaging of cytotoxic cargo; the ‘*in vivo* persistence’ advantage described for infused cells is not the primary rationale for a cell-free EV product. (3) Checkpoint ablation: Checkpoint edits (e.g., PD-1 deletion) are most directly relevant to living NK-cell therapies. For an EV product, their most plausible relevance is indirect—by preventing inhibitory programming of producer cells during prolonged culture/conditioning, which may secondarily influence EV composition; direct evidence for improved EV potency requires head-to-head comparisons of edited vs unedited producer lines.

### 5.3 Surface display engineering: improving lung tumor homing

While upstream editing endows biological activity, surface engineering of exosomes focuses on physical targeting and bioavailability. EVs may incorporate selected membrane proteins from their producer cells, but incorporation is selective rather than automatic and may vary with protein topology, intracellular trafficking, producer-cell state, and EV biogenesis. Surface display of CAR, hnCD16, membrane-bound cytokine complexes, or other ligands must therefore be confirmed and quantified at the EV level (104). (1) CAR-mediated homing: Exosomes derived from CAR-NK cells (CAR-NK-Exos) display the CAR construct, which facilitates specific binding to tumor cells. In the EV context, the CAR is best viewed as a surface targeting module (via its extracellular scFv), whereas intracellular signaling domains (e.g., CD3ζ, CD28/4-1BB) are likely functionally inert on EVs and primarily reflect the parental CAR design. In selected HER2-positive models, CAR-displaying EV preparations showed antigen-associated binding or tumor accumulation. These observations were model- and labeling-dependent and should not be generalized as evidence of precise, uniform, or clinically sufficient tumor homing (22). (2) Dual-targeting ligands: Beyond CARs, exosomes can be engineered to display additional homing peptides or ligands. Kang et al. utilized a surface-display system to anchor anti-EGFR antibodies (cetuximab) and IL-15 on the surface of NK-derived vesicles. This approach illustrates an alternative to full-length CAR display: smaller antibody- or scFv-based ligands fused to a membrane anchor may also be used for EV targeting when the primary objective is tumor-cell engagement. These dual-engineered EVs showed preferential uptake by EGFR-positive lung cancer cells and induced significantly greater apoptosis than unmodified controls (105). (3) Ligand-receptor optimization: Strategies such as overexpressing NKG2D and IL-24 on the exosome surface have also been reported to enhance uptake by A549 lung cancer cells, triggering TRAIL-mediated apoptotic pathways.

### 5.4 Cargo loading enriches EV/sEVs with cytotoxic or regulatory payloads

**Figure 4:**
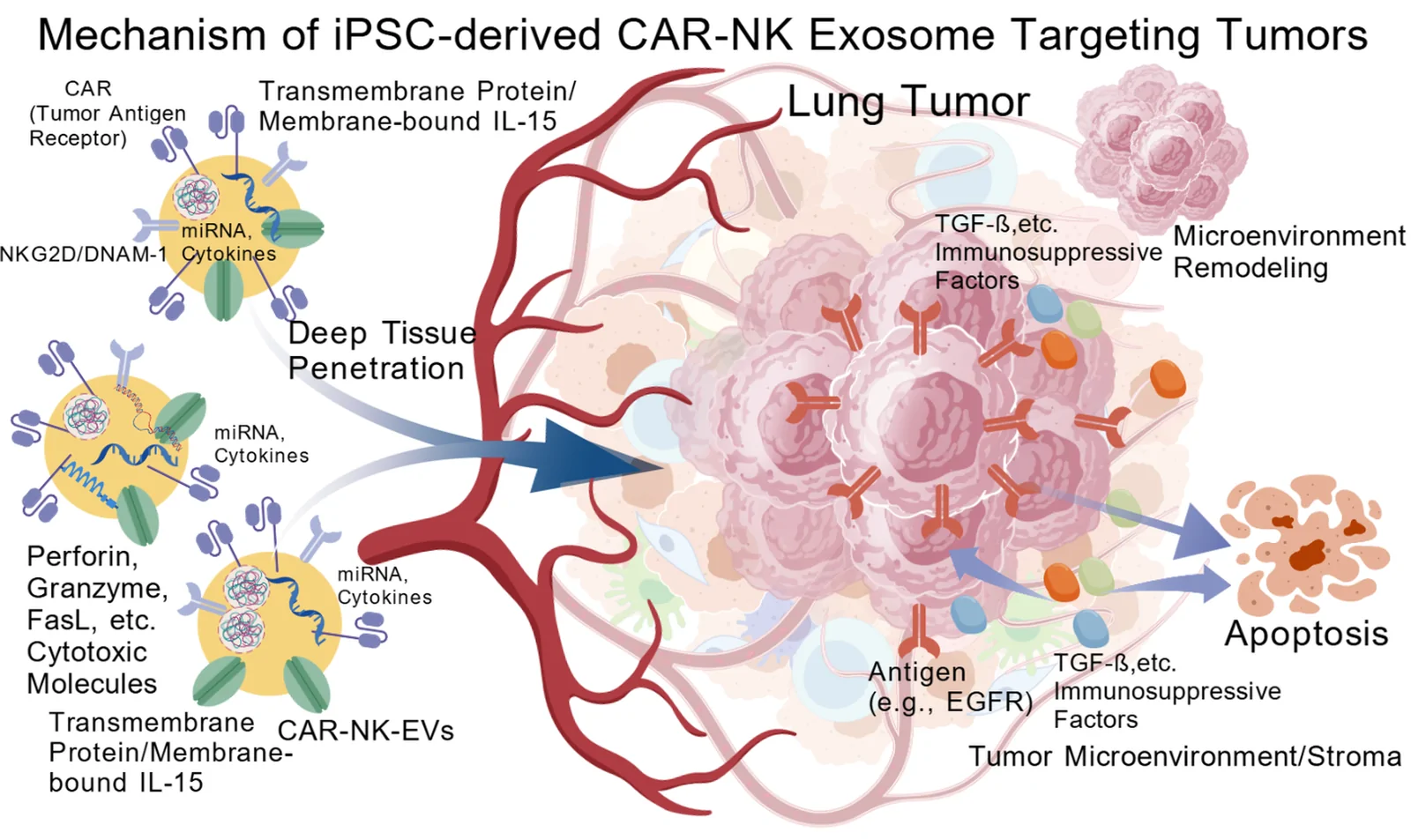
Proposed mechanism of iPSC-derived CAR-NK EV/sEV targeting in lung tumors. Engineered CAR-NK-derived EVs/sEVs are proposed for local or systemic delivery to lung tumors; however, deposition in the lung does not necessarily indicate exposure of tumor cells. Conducting-airway mucus, mucociliary clearance, regional ventilation, pulmonary macrophage uptake, stromal heterogeneity, and variable antigen density may each limit the fraction of intact and functionally active vesicles reaching individual tumor regions. If CAR-positive vesicles retain surface targeting modules after formulation and aerosolization, EV/sEV surface ligands may support antigen-dependent tumor engagement. Following binding and uptake, vesicles may deliver cytotoxic or regulatory cargo, provided that cargo integrity and functional potency are retained after manufacturing, storage, and pulmonary delivery. Created with BioGDP.com.

The luminal cargo of exosomes can be enriched with cytotoxic or modulatory agents through both biological priming and physicochemical loading techniques (106). (1) Endogenous cargo enrichment: Cytokine priming of the producer NK cells significantly alters exosome composition (43). Pre-treatment with IL-15 and IL-21 has been shown to upregulate the packaging of Granzyme B, Perforin, and FasL into secreted vesicles, enhancing their lytic potency against solid tumors (26, 90). Hypoxic conditioning has been associated with altered NK-EV cargo in selected experimental systems, but whether these changes improve antitumor activity, introduce unwanted stress-associated cargo, or remain reproducible after manufacturing scale-up requires comparative profiling and functional testing. (2) Exogenous chemotherapy loading: The lipid bilayer of exosomes permits the loading of hydrophobic drugs (107). Drug-loaded NK-EV preparations have shown enhanced activity relative to free drug in selected preclinical models. However, loading efficiency, unencapsulated drug carryover, vesicle integrity, aggregation, release kinetics, and true dose equivalence were not consistently controlled across studies, limiting cross-study interpretation. (3) Nucleic acid delivery: Non-iPSC NK-EV preparations have been explored as nucleic-acid carriers, but direct evidence that iPSC-derived CAR-NK EV/sEVs provide efficient, reproducible, and safe nucleic-acid delivery is not currently available. Studies have successfully loaded siRNA targeting *BCL-2* or *GPRC5A* into NK-exosomes, leading to the silencing of survival genes and sensitization of ovarian and lung cancer cells to chemotherapy (108). Furthermore, enrichment with tumor-suppressive miRNAs, such as miR-186 or let-7b, allows these vesicles to reverse immune suppression and inhibit cancer cell proliferation (45) (**Figure 4**).

## 6 Applications and translational challenges of iPSC-CAR-NK EV/sEVs in lung cancer

NSCLC develops within a highly compartmentalized organ in which conducting airways, distal alveoli, interstitial tissue, pulmonary vasculature, and draining lymphatic structures have distinct epithelial and immune-cell compositions. Consequently, the barriers encountered by an EV product depend not only on tumor biology but also on tumor location, administration route, regional ventilation, airway patency, mucus properties, and the distribution of pulmonary phagocytes. These lung-specific variables distinguish NSCLC delivery from generic solid-tumor EV delivery and should be incorporated into product design and nonclinical testing (98). Similarly, physical barriers – a stiff extracellular matrix and irregular vasculature – impede immune cell and drug penetration into solid tumors. These features – stromal shielding, intratumoral heterogeneity, and active immune evasion – have made lung cancers notoriously hard to treat with any therapy, including cellular immunotherapy. Thus, lung cancer represents an important disease context in which to evaluate novel immune-based approaches, including iPSC-derived CAR-NK EV/sEV products.

### 6.1 Lung-specific barriers to EV/sEV delivery: pulmonary immune architecture, mucus clearance, stromal diversity, and antigen heterogeneity

The lung is organized into anatomically and immunologically distinct conducting-airway, interstitial, and alveolar compartments. Inhaled EVs deposited in proximal airways encounter mucus, ciliated epithelium, airway dendritic cells, and macrophages, whereas particles reaching distal alveoli encounter alveolar epithelial cells and resident alveolar macrophages. Rapid uptake by pulmonary phagocytes may reduce the fraction of intact vesicles available to engage tumor cells, although such uptake could also contribute to local immunomodulation. Total lung deposition should therefore not be interpreted as tumor-specific exposure (98). Airway mucus and mucociliary transport constitute active clearance mechanisms rather than passive diffusion barriers. EV retention is expected to depend on mucus mesh structure, surface charge, aggregation state, ciliary function, airway obstruction, and regional ventilation, all of which may vary with smoking-related airway disease, infection, prior thoracic treatment, tumor location, and tumor-associated bronchial obstruction. Pulmonary formulations should therefore be evaluated in mucus-containing airway models and disease-relevant lung models, with direct measurement of aerosol stability, mucus penetration, clearance kinetics, and intact-vesicle recovery (109). NSCLC stroma is not spatially uniform. Single-cell analyses of human lung tumors have identified diverse fibroblast, endothelial, myeloid, and lymphoid states, while multiregion studies of lung adenocarcinoma have demonstrated progressive changes from distant normal lung to tumor-adjacent tissue and established tumor. Collagen organization, vascular permeability, hypoxia, macrophage abundance, and immune exclusion may therefore differ between the tumor edge and core, among separate lesions, and between primary and metastatic sites. Nanoscale size alone is insufficient to predict uniform penetration, and EV activity should be evaluated in spatially resolved models that preserve subtype-specific stromal architecture (110). Antigen selection must account for histologic, molecular, spatial, and temporal heterogeneity. Candidate targets such as EGFR, HER2, mesothelin, B7-H3, and CEACAM5 are not uniformly expressed across lung adenocarcinoma and squamous-cell carcinoma, and target abundance may differ between biopsy and resection specimens, primary tumors and metastases, or pretreatment and post-treatment samples. Multiregion and longitudinal studies also demonstrate substantial clonal divergence within NSCLC. Eligibility based on a single biopsy may therefore overestimate the proportion of target-positive disease, and future programs should define quantitative antigen-density and tumor-coverage thresholds using multiregion sampling when feasible. These lung-specific barriers support separate evaluation in lung adenocarcinoma and squamous-cell carcinoma models, use of orthotopic and immunocompetent systems in addition to subcutaneous xenografts, and distinct assessment of central airway tumors, peripheral parenchymal lesions, and metastatic sites. Multispecific targeting may reduce single-antigen escape but would increase product-complexity, potency-assay, and normal-tissue safety requirements (111).

### 6.2 Preclinical NK-EV and CAR-EV studies provide indirect support for lung-cancer translation

Preclinical studies provide indirect support for NK-EV activity in lung-cancer models, including engineered NK-92-derived EVs and patient-derived NK-EVs evaluated in xenografts, organoids, or *in vitro* systems. Strengths of this literature include the use of three-dimensional tumor models, antigen-directed engineering in selected studies, and demonstration of biological activity beyond two-dimensional cytotoxicity assays. However, the evidence base is heterogeneous: producer-cell sources, culture conditions, isolation methods, EV characterization, dose units, tumor models, and comparator arms differ substantially, and some studies cannot exclude contributions from non-vesicular proteins, residual cytokines, or unencapsulated therapeutic cargo. No study has directly tested iPSC-derived CAR-NK EV/sEVs in NSCLC. Evidence from living iPSC-NK cells supports producer-cell manufacturing and cellular antitumor feasibility, whereas evidence from CAR-NK EVs in non-iPSC or non-NSCLC models supports EV-level targeting plausibility. Neither evidence stream alone demonstrates that iPSC-derived CAR-NK EVs will retain CAR display, cytotoxic cargo, tumor accumulation, or antitumor efficacy in lung cancer. These findings therefore justify direct comparative experiments rather than an expectation of therapeutic efficacy (44, 112).

### 6.3 Reprogramming the tumor neighborhood

A potential feature of NK-derived EVs is the concurrent transfer of cytotoxic and immunomodulatory cargo, but the direction and magnitude of these effects are cargo-, dose-, recipient-cell-, and microenvironment-dependent. This multifunctionality is not uniformly beneficial: EV preparations may contain both pro-inflammatory and immunoregulatory mediators, and changes in producer-cell activation or purification may alter their relative abundance. Consequently, immune modulation should be evaluated bidirectionally, including unintended suppression, excessive inflammation, and effects on non-tumor immune cells. NK‐EVs naturally carry cytokines (IL-10, TNF-α, IFN-γ), chemokines (CCL3/4/5), and even growth factors that act on immune cells (113). When these EVs are taken up by tumor or stromal cells, they can deliver IFN-γ to inhibit angiogenesis and sensitize cells to apoptosis (114), and TNF-α to trigger tumor death pathways (115). Crucially, NK‐EVs also deliver miRNAs that reprogram suppressive signals. For instance, NK‐EV‐borne miR-186 targets TGF-β receptors in neuroblastoma cells and relieves TGF-β‐mediated inhibition of NK cells (116). In lung cancer models, miRNA‐loading of NK‐EVs (e.g. miR-5193/miR-149-5p) downregulated the PD-1/PD-L1 axis and oncogenic FOXM1, reversing checkpoint‐driven escape (117). In an NSCLC xenograft model, the combination of miRNA-enhanced NK-EVs and carboplatin produced greater tumor suppression than carboplatin alone and was associated with increased apoptosis involving SOD2/PARP signaling and inhibition of NF-κB/TGF-β-related inflammatory pathways (117, 118). Beyond soluble factors, NK EVs also “educate” immune cells. NK‐EVs upregulate HLA‐DR and co‐stimulatory molecules on monocytes and dendritic cells, activating them to prime T cells (113, 119). They can skew macrophages to an M1 (inflammatory) phenotype and suppress M2 polarization. In one lung‐injury model, NK EV treatment drove macrophages toward M1 and dramatically reduced neutrophil infiltrates, hinting that such EVs could break the immunosuppressive myeloid environment in tumors (120). Furthermore, CAR‐modifications on EVs can add new tools to reprogram the niche. For example, exosomes engineered with the transferrin receptor‐binding peptide (T7) and an anti‐HER2 CAR penetrated the blood–brain barrier and delivered a photosensitizer/ferroptosis inducer to brain tumors (22). These findings suggest that displaying a CAR or another targeting ligand on an EV may improve cargo delivery across selected biological barriers; however, the efficiency and precision of this process remain model- and product-dependent. Accordingly, tumor-microenvironment modulation should be treated as a product-specific and experimentally testable mechanism rather than as an inherent property of all CAR-NK EVs. Studies should distinguish direct tumor-cell killing from effects on macrophages, dendritic cells, T cells, and other recipient populations and should evaluate both antitumor and potentially counterproductive immune responses.

### 6.4 Combination strategies may enhance CAR-NK EV/sEV activity in NSCLC

Combination strategies involving immune-checkpoint blockade, chemotherapy, radiotherapy, or targeted therapy are biologically plausible but remain hypothesis-generating. Reported enhancement has largely been observed with non-iPSC NK-EV systems in selected preclinical models, and many studies did not include formal interaction analyses, optimized sequence or timing comparisons, dose-equivalent controls, or comprehensive toxicity assessments. Future studies should compare each monotherapy with the combination at matched exposures, determine schedule dependence, and establish whether any added efficacy reflects EV-specific biological interaction rather than increased total therapeutic exposure. Combination efficacy should also be evaluated in antigen-heterogeneous, immunocompetent, and treatment-resistant NSCLC models. Until these data are available, combination approaches should be presented as rational candidates for testing rather than as an expected clinical treatment paradigm (112, 118) (**Table 6**).

**Table 6:** Numeric claims and source evidence relevant to iPSC-CAR-NK EV/sEV translation.

Numeric claim	Context / population / model	Product or platform	Route / dose / regimen	Source type	Primary citation	Limitation	Directly relevant to iPSC-CAR-NK EVs?
NSCLC accounts for approximately 85% of lung cancer cases	Epidemiology of lung cancer	NSCLC	Not applicable	Peer-reviewed epidemiology / cancer statistics	Bray et al., 2024 (2)	General disease background; not EV-specific	No
86 patients treated with FT596	Phase 1 trial in relapsed/refractory B-cell lymphoma	iPSC-derived CD19 CAR-NK cell therapy	Lymphodepletion followed by FT596 with or without rituximab	Peer-reviewed clinical trial	Ghobadi et al., 2025 (23)	Hematologic malignancy; living-cell product; not EV/sEV	No; clinical analog only
RP2D preliminarily identified as 1.8 × 10^9 cells, given as three doses per cycle	FT596 phase 1 trial	iPSC-derived CD19 CAR-NK cell therapy	Three FT596 doses per cycle after lymphodepletion	Peer-reviewed clinical trial	Ghobadi et al., 2025 (23)	Cell dose cannot be converted into EV dose; does not define EV particle number or CAR-positive EV dose	No; informs manufacturing scale only
CRS in 1/18 patients in regimen A and 9/68 patients in regimen B; no neurotoxicity observed	FT596 phase 1 trial safety	iPSC-derived CD19 CAR-NK cell therapy	Regimen A without rituximab; regimen B with rituximab	Peer-reviewed clinical trial	Ghobadi et al., 2025 (23)	Safety data apply to living cells in lymphoma, not EVs in NSCLC	No; safety analog only
"85% complete remission rate"	Previously stated in manuscript	FT596 or related iPSC-CAR-NK clinical data	Not clearly specified	Unclear / requires verification	Should be replaced or fully sourced	Denominator, regimen, disease subgroup, endpoint, and source are unclear; should not be stated without exact citation	No
Approximately 1000-fold expansion in stirred bioreactors	iPSC-derived NK-cell manufacturing study	iPSC-derived NK cells	Repeated stimulation/expansion; scale-up from 1 L to 10 L aerated stirred bioreactors	Peer-reviewed or indexed manufacturing study	Kikuchi et al., 2025 (57)	Cell expansion study; not EV production; does not establish EV yield or potency	Indirect; manufacturing relevance
>10^13 NK-derived EVs per batch	Large-scale NK and NK-EV biomanufacturing workflow	NK-cell-derived EVs	GMP-like or clinically relevant hollow-fiber / large-scale workflow	Peer-reviewed EV manufacturing study	St-Denis-Bissonnette et al., 2023 (58)	NK-EV product, not iPSC-CAR-NK EV; potency and dose equivalence to CAR-NK EV unknown	Indirect; EV manufacturing relevance
Approximately 38-fold higher EV harvest compared with planar flask culture	Large-scale bioreactor production	MSC-derived EVs	Hollow-fiber bioreactor vs flask culture	Peer-reviewed EV manufacturing study	Kink et al., 2024 (59)	MSC source, not NK or iPSC-NK; cannot be assumed for CAR-NK EVs	Indirect; manufacturing analogy only
TFF provides higher EV recovery than ultracentrifugation	Downstream EV concentration and purification	EVs from large fluid volumes	Tangential flow filtration compared with ultracentrifugation	Peer-reviewed methods study	Busatto et al., 2018 (62)	If a specific fold-change such as "140-fold" is stated, it must be verified against the primary article and exact experimental condition	Indirect; downstream processing relevance
100 μg/kg weekly for two weeks	Published MSC-exosome clinical study in chronic kidney disease summarized in clinical review	Umbilical cord blood MSC-derived exosomes	First dose intravenous; second dose intra-arterial	Peer-reviewed review summarizing clinical study	Lotfy et al., (2023) (121)	Non-oncology, MSC-derived product; protein-mass dose, not particle number or CAR-positive EV dose	No; dose-format precedent only
8.5 × 10^11 to 4.0 × 10^13 exosomes / MHC class II-positive particles per injection	Dendritic-cell-derived exosome cancer vaccine trials	DC-derived exosomes	Injection-based immunotherapy	Peer-reviewed review / clinical trial summary	Chen et al., 2020 (122); Besse et al., 2016 (123)	DC vaccine product, not cytotoxic CAR-NK EV; dose unit may reflect MHC-II molecule or particle-associated reporting	No; clinical EV dose precedent only
Inhaled PTX@CAR-Exos accumulated in tumor area, reduced tumor size, and prolonged survival	Orthotopic or in situ lung cancer mouse model	Paclitaxel-loaded CAR-T cell-derived EVs	Inhalation delivery	Peer-reviewed preclinical study	Zheng et al., 2023 (94)	CAR-T EV, drug-loaded product, mouse model; not iPSC-CAR-NK EV; aerosol stability and human deposition remain unresolved	Indirect; inhalation and lung-cancer model relevance
10^12–10^13 EVs per administration and 10^13–10^14 particles per treatment course	Manufacturing demand estimate in manuscript	Hypothetical therapeutic EV product	Repeat dosing; route unspecified	Author extrapolation	Should be labeled as extrapolation	Not an established clinical dose; depends on potency, route, CAR-positive EV fraction, and tumor exposure	No; hypothesis only
10^8–10^9 particles daily for aerosol regimens	Previously stated inhalation dosing precedent	MSC-EV or other inhaled EV clinical/registry examples	Aerosol or nebulized route	Registry / clinical review / non-peer-reviewed depending on source	Must specify exact trial/source	Do not generalize to CAR-NK EV; particle-count units vary across studies	No; route precedent only
40–45% reduction in NSCLC organoid viability	Patient-derived or autologous NK-EV NSCLC organoid study	NK-cell-derived EVs	In vitro organoid exposure	Peer-reviewed preclinical / translational study	Palade et al., 2025 (98)	Not CAR-engineered; not iPSC-derived; in vitro organoid model	Indirect; NSCLC NK-EV relevance
Enhanced killing or tumor suppression in EGFR-positive lung cancer models	Surface-engineered NK-sEV study	NK-92-derived sEVs with EGFR-targeting antibody and IL-15	In vitro and mouse lung cancer models	Peer-reviewed preclinical study	Kang et al., 2025 (96)	Not CAR-NK; NK-92 source; surface-antibody engineering differs from CAR display	Indirect; lung-cancer engineered NK-sEV relevance

Quantitative claims are listed with their source model or population, product platform, dose unit, regimen, evidence type, primary citation, limitation, and relevance to iPSC-derived CAR-NK EV/sEVs. Cell-therapy data, MSC-EV doses, dendritic-cell exosome doses, CAR-T EV studies, and NK-EV manufacturing outputs are considered clinical-analog or indirect evidence unless they directly test iPSC-derived CAR-NK EVs/sEVs in NSCLC. Hypothetical particle-dose estimates are labeled as capacity-planning assumptions. EV terminology follows MISEV2018.

## 7 Clinical development landscape and evidence boundaries

Direct clinical evidence for iPSC-derived CAR-NK EV/sEV therapy in NSCLC is not yet available (124). Therefore, living iPSC-NK and CAR-NK cell trials should be interpreted as clinical analogs rather than as evidence of EV efficacy, safety, or dosing (23, 125). Their main relevance is to three translational questions: whether clonal NK-lineage products can be manufactured at clinical scale, whether multiplex CAR-NK engineering can be advanced into humans, and which safety endpoints should be prioritized during early EV-product development (126). The most relevant iPSC-derived clinical analog is FT596, a CD19-directed iPSC-CAR-NK product generated from a clonal master iPSC line and engineered to express a CAR, high-affinity non-cleavable CD16, and an IL-15/IL-15Rα fusion module. In a phase 1 trial involving patients with relapsed or refractory B-cell lymphoma, FT596 demonstrated the feasibility of off-the-shelf, repeat-dose iPSC-CAR-NK-cell administration, with a preliminary recommended phase 2 dose of 1.8 × 10^9^ cells administered in three doses per cycle. Its reported safety profile, including low-grade CRS and absence of observed neurotoxicity, supports the clinical tractability of iPSC-derived CAR-NK cells in selected hematologic settings (127). Other CAR-NK studies, including cord-blood-derived CD19- or CD33-targeted CAR-NK products, similarly suggest that CAR-NK cells can be administered with manageable early safety profiles in small clinical cohorts, although efficacy and durability remain context-dependent and require further validation (128, 129). For iPSC-CAR-NK EV/sEV development, the inference from these living-cell trials should remain deliberately limited (130). Cell-therapy data can inform source-cell standardization, engineering feasibility, dose-scale awareness, repeat-dose logistics, and safety-monitoring priorities, but they cannot define EV pharmacokinetics, biodistribution, tumor exposure, immunogenicity, potency units, or pulmonary delivery behavior (131). Modules that are functional in living CAR-NK cells, including CARs, hnCD16, IL-15 support, or suppression-resistance edits, may influence EV composition or surface display; however, their incorporation, abundance, and bioactivity must be verified directly at the vesicle level (112). Thus, the next critical step is not further extrapolation from iPSC-NK or CAR-NK cell trials, but standardized benchmarking of iPSC-NK-derived EVs against primary NK-EVs, CAR-negative EVs, and unmodified NK-cell-line EVs under matched culture, isolation, and potency-assay conditions (132). Such comparisons should integrate CAR-positive vesicle fraction, CAR density, cytotoxic cargo, multi-omics cargo profiling, antigen-dependent uptake, dose-normalized cytotoxicity, biodistribution, and repeat-dose safety. Only after these EV-specific parameters are defined can clinical analog evidence from iPSC-NK and CAR-NK cell therapies be translated into a rational development path for iPSC-CAR-NK EV/sEV products in NSCLC (133) (**Table 7**).

**Table 7:** Clinical development landscape relevant to iPSC-derived CAR-NK EV/sEV translation.

Platform/product	Product type and key engineering	Indication	Study identifier/phase	Development status at data cutoff	Key publicly available information	Relevance to iPSC-CAR-NK EV/sEV development	Principal limitation
NK-cell-derived EV therapeutic products	EV/sEV preparations produced by primary NK cells or NK-cell lines	Cancer	No registered therapeutic study identified	No registered interventional clinical study identified	Published studies remain preclinical, including in vitro, organoid, and animal-model investigations	Represents the closest biological analog for endogenous NK cytotoxic cargo	No human dose, PK, biodistribution, safety, or efficacy data; products and manufacturing methods remain heterogeneous
CAR-engineered EV therapeutic products	EVs displaying CAR-derived targeting modules from CAR-T, CAR-NK, or other engineered producer cells	Cancer	No registered therapeutic study identified	No registered interventional clinical study identified	Published evidence is limited to preclinical antigen-binding, cytotoxicity, cargo-delivery, and tumor-model studies	Provides mechanistic support for EV-level CAR display and antigen-dependent targeting	No clinical evidence; CAR-positive EV fraction, active dose, biodistribution, and safety remain undefined
FT516	Clonal iPSC-derived NK cells expressing high-affinity non-cleavable CD16; non-CAR cellular product	Relapsed or refractory B-cell lymphoma	NCT04023071; phase 1	Completed; peer-reviewed results published	Fifty-five patients received FT516; no dose-limiting toxicities were reported, one grade 1 CRS event occurred, and neurotoxicity was not observed	Supports clonal iPSC-NK manufacturing, cryopreservation, repeat dosing, antibody-combination logic, and clinical safety monitoring	Living-cell and non-CAR product; cannot define EV-level hnCD16 display, EV dose, PK, or tumor exposure
FT596	Clonal iPSC-derived CAR-NK cells expressing a CD19 CAR, hnCD16, and an IL-15/IL-15Rα fusion module	Relapsed or refractory B-cell lymphoma	NCT04245722; phase 1	Completed and published; subsequent development was discontinued/superseded	The completed first-in-human study reported safety, pharmacokinetic, dose-escalation, and antitumor-response data	Strong clinical analog for multiplex iPSC-CAR-NK engineering, repeat-dose administration, and cell-product manufacturing	Hematologic indication and living-cell product; does not establish EV CAR display, EV dose, biodistribution, or NSCLC activity
CNTY-101	iPSC-derived CD19 CAR-NK cells incorporating allo-evasion, IL-15 support, and a safety switch	Relapsed or refractory CD19-positive B-cell malignancies	NCT05336409; phase 1	Ongoing; active, not recruiting	Early clinical reports address safety, cellular pharmacokinetics, allo-evasion, and preliminary responses	Supports clinical evaluation of extensively edited clonal iPSC-CAR-NK products	Early-stage, living-cell and hematologic-malignancy evidence; no direct relevance to EV pharmacology
FT522 follow-on development	iPSC-derived CD19 CAR-NK cells incorporating hnCD16, cytokine support, CD38 disruption, and an alloimmune-defense receptor	B-cell malignancies and proposed B-cell-mediated autoimmune indications	NCT05950334 for the lymphoma study; additional development described by the sponsor	Clinical data reported for the lymphoma program; further development planned or under evaluation	Sponsor reports describe continued assessment of the platform and planned development in additional indications	Illustrates evolving allo-evasion and reduced-conditioning strategies that may inform producer-cell engineering	Sponsor-reported plans may change; not an EV product and not direct evidence for NSCLC
Proposed iPSC-derived CAR-NK EV/sEV product for NSCLC	EV/sEV-enriched product proposed to display CAR and contain NK-associated cytotoxic cargo	NSCLC	No registry identifier	Preclinical concept; no clinical study initiated	No human data are available	Defines the product-specific clinical evidence gap addressed by this review	Requires direct product characterization, potency standardization, PK/biodistribution, toxicology, GMP manufacture, and regulatory authorization before first-in-human testing

The table distinguishes therapeutic EV products from living iPSC-derived NK and CAR-NK cell therapies and categorizes programs as completed, ongoing, planned development, or no registered interventional study identified. Study status was assessed using publicly available registry records, peer-reviewed publications, and sponsor-reported information with a data cutoff of July 31, 2026. Sponsor-announced programs without confirmed enrollment are classified as planned development rather than as ongoing clinical trials. Observational studies measuring EV biomarkers after living-cell therapy are not included. None of the listed living-cell studies constitutes direct clinical evidence for iPSC-derived CAR-NK EV/sEV therapy in NSCLC.

## 8 Current challenges and future perspectives

### 8.1 EV heterogeneity and definition of the active product fraction

The central product-development challenge is that an iPSC-CAR-NK EV/sEV product is not a single molecular entity but a heterogeneous vesicle-enriched preparation. Even when producer cells originate from one clonal iPSC bank, released vesicles may differ in size, biogenesis, surface CAR density, NK-associated proteins, cytotoxic cargo, and RNA content, while the final preparation may also contain non-vesicular proteins, lipoproteins, or residual soluble factors. Consequently, total particle number or bulk protein concentration may not accurately represent the therapeutically active fraction. Product development should therefore quantify the CAR-positive vesicle fraction, CAR density, cargo distribution, relevant impurities, and the relationship of these attributes to functional potency (134).

### 8.2 Mononuclear phagocyte system clearance, biodistribution, and limited tumor exposure

Pharmacokinetics and tumor exposure constitute major barriers to the systemic use of iPSC-derived CAR-NK EV/sEV products. After intravenous administration, unmodified EV preparations may disappear rapidly from the blood and become enriched in the liver, spleen, and, depending on particle characteristics and route, the lungs. Experimental macrophage depletion delays systemic EV clearance, indicating that Kupffer cells, splenic macrophages, circulating monocytes, and other phagocytic populations contribute substantially to the removal of administered vesicles. These findings imply that the administered dose, circulating dose, and tumor-accessible dose may differ by several orders of biological relevance even when they are reported using the same particle-number unit (52). Organ-level signal should not automatically be interpreted as intact and functionally active EV accumulation. Lipophilic dyes, detached radionuclides, degraded reporter proteins, or labeled non-vesicular contaminants may persist after vesicle disruption and thereby overestimate EV retention. Biodistribution studies should therefore use at least two orthogonal approaches where feasible, distinguish intact vesicles from free label or degradation products, and report blood clearance, tissue concentration, tumor-to-liver and tumor-to-spleen ratios, cellular localization, and recovery of functional activity from relevant tissues. Single-time-point whole-organ fluorescence is insufficient for defining EV pharmacokinetics (135). Surface engineering represents one possible strategy to reduce phagocytic clearance. PEG-containing surface modifications have prolonged EV circulation in mice, while CD47-dependent antiphagocytic signaling and albumin-binding domains have also increased systemic exposure in selected models. However, these approaches cannot be assumed to improve tumor delivery proportionally. Surface shielding may mask CAR or other targeting ligands, alter cellular uptake, introduce additional critical quality attributes, and affect lot comparability. The optimum surface-engineering strategy should therefore be selected according to tumor-to-liver and tumor-to-spleen exposure rather than blood half-life alone (136). CD47-based engineering requires particular caution in an anticancer product. Although EV-associated CD47 may reduce macrophage uptake and prolong circulation, CD47 displayed or transferred by EVs can inhibit phagocytosis. Any circulation advantage must therefore be evaluated against the possibility of suppressing macrophage-mediated clearance of tumor cells or altering the function of hepatic, splenic, and tumor-associated macrophages. CD47 density, retention on formulated EVs, transfer to recipient cells, systemic immune effects, and antitumor consequences should be evaluated together rather than treating CD47 solely as a pharmacokinetic module (137). Tumor-targeting ligands, including CARs, antibodies, nanobodies, or homing peptides, may increase binding after a vesicle reaches the tumor but do not necessarily prevent first-pass hepatic or splenic sequestration. Targeting studies should therefore include matched non-targeted EVs and quantify both absolute tumor exposure and tumor-to-normal-organ distribution. Similarly, inhaled or intratracheal administration may increase local lung exposure but does not establish delivery to tumor cells because EVs may remain in mucus, normal parenchyma, airway macrophages, or alveolar macrophages. Route optimization should compare systemic and pulmonary administration using the same product, dose unit, labeling method, and pharmacodynamic endpoint (25). Pharmacological depletion, inhibition, or saturation of the mononuclear phagocyte system can increase nanoparticle or EV circulation in experimental animals, but such approaches deliberately perturb systemic phagocyte function and are less directly transferable to repeated treatment of patients with cancer. They are more appropriately used as mechanistic tools unless an acceptable safety margin is established. Consequently, the preferred development strategy should integrate product-level surface engineering, route and formulation optimization, and dose scheduling before considering systemic macrophage manipulation (52).

### 8.3 Variability in cargo loading and dose definition

Cargo loading is intrinsically variable. Endogenous loading depends on producer-cell differentiation, activation, metabolic state, and culture conditions, whereas exogenous loading methods may alter membrane integrity, aggregation, cargo release, or the amount of unencapsulated material. A dose defined only by total particle number may therefore contain variable quantities of CAR, granzyme B, perforin, FasL, RNA, cytokine complexes, or therapeutic drug. Loading efficiency, encapsulated versus free cargo, cargo distribution across vesicle subpopulations, release kinetics, and dose-normalized functional activity should be included in process characterization and lot-comparability testing. Particle count, bulk protein, and cargo mass should be treated as complementary rather than interchangeable dose metrics until a validated potency-linked unit is established (90, 138).

### 8.4 Repeat-dose immunogenicity and safety

Repeated administration may introduce immunological risks that are not predicted by single-dose studies. Potential contributors include the CAR-derived scFv, engineered cytokine or Fc-binding modules, producer-cell proteins, altered surface glycans, residual host-cell material, vector-related impurities, and process-derived contaminants. Repeated exposure could induce anti-product antibodies, complement activation, cytokine responses, altered phagocytic clearance, tissue inflammation, or progressive loss of biological activity. Repeat-dose studies should therefore assess humoral and cellular immune responses, complement activation, cytokine release, changes in pharmacokinetics, organ-specific inflammation, and preservation of antitumor activity. These evaluations should include immunocompetent models and human immune-cell assays because severely immunodeficient xenograft models are insufficient for evaluating immunogenicity (139).

### 8.5 Manufacturing reproducibility and potency bridging

Manufacturing reproducibility remains a distinct challenge despite use of a clonal iPSC source. Variation can arise during iPSC expansion, NK-cell differentiation, producer-cell activation, EV harvest, purification, concentration, formulation, freezing, thawing, and aerosolization. Source-cell standardization reduces donor variability but does not guarantee consistent EV biogenesis, cargo packaging, or surface composition.

Critical process parameters should be linked to critical quality attributes and to a mechanism-based potency matrix across multiple independent lots. Comparability plans are required for changes in the master cell bank, medium, differentiation protocol, culture scale, bioreactor, purification process, formulation, storage conditions, or pulmonary delivery device. A release assay based solely on particle count, marker expression, or short-term *in vitro* cytotoxicity would be insufficient unless it predicts *in vivo* exposure and antitumor activity. Future manufacturing reports should include overall recovery, potency-adjusted yield, CAR-positive vesicles per batch, labor and consumable burden, release-test turnaround time, failed-batch frequency, storage-associated losses, and estimated clinical doses per batch. These parameters are required to distinguish technical scale-up from commercially relevant scale-up. A commercially viable process must also demonstrate that the analytical and release-testing burden does not dominate production cost or delay product release beyond the validated shelf-life window (59, 75).

### 8.6 Priority experiments

The next development milestone should be direct testing of iPSC-derived CAR-NK EV/sEVs in NSCLC models using matched CAR-negative EVs, antigen-negative tumor controls, antigen-blockade experiments, dose-normalized comparisons, rigorous EV characterization, quantitative biodistribution, and repeat-dose safety assessment. Head-to-head comparisons with primary NK-EVs, NK-cell-line EVs, and living iPSC-CAR-NK cells would clarify which properties are attributable to the iPSC source, CAR display, EV format, or their combination. Until these data are available, the platform should be described as biologically plausible but experimentally unvalidated rather than as an established therapeutic platform (44).

## 9 Conclusions

Evidence from NK-cell-derived EVs, CAR-engineered EVs, and living iPSC-NK or CAR-NK products supports the biological plausibility of an iPSC-derived CAR-NK EV/sEV platform. However, no study has yet demonstrated the complete platform in NSCLC, and evidence from its individual components cannot be assumed to be additive or directly transferable. The strongest current evidence concerns the feasibility of clonal iPSC-NK producer-cell manufacturing and the cytotoxic activity of selected NK-EV preparations. Major unresolved issues include reproducible CAR display, definition of the active vesicle fraction, rapid hepatic and splenic sequestration by the mononuclear phagocyte system, limited and heterogeneous tumor exposure, repeat-dose immunogenicity, manufacturing comparability, and the absence of a potency assay validated against *in vivo* or clinical activity. Proposed circulation-extension strategies, including PEG-containing modifications, CD47-mediated phagocytosis avoidance, albumin binding, targeting ligands, or local pulmonary delivery, remain product-specific and may introduce additional biological, safety, and CMC trade-offs.

FDA, the European Union regulatory network, and ISEV have complementary but distinct roles. FDA regulates therapeutic exosome products as drugs and biological products; the European Union applies a product-specific medicinal-product or ATMP classification and the corresponding GMP framework; and ISEV provides non-binding scientific recommendations for rigorous EV studies. Formal regulatory classification and GMP expectations therefore cannot be inferred from compliance with MISEV recommendations alone. Clinical progression should follow a stage-gated pathway comprising product and mechanism definition, standardized potency, reproducible GMP-compatible manufacturing, formulation and stability qualification, quantitative pharmacokinetic and biodistribution studies, disease-relevant efficacy, repeat-dose toxicology and immunogenicity, and jurisdiction-specific regulatory interaction. First-in-human investigation should begin only when the clinical product is analytically defined, the intended administration route is supported by exposure data, and the starting dose and monitoring plan are justified by an integrated nonclinical package. At present, iPSC-derived CAR-NK EV/sEVs should be positioned as an investigational translational strategy rather than a clinically validated therapy for NSCLC.
